# Hypertoxic self-assembled peptide with dual functions of glutathione depletion and biosynthesis inhibition for selective tumor ferroptosis and pyroptosis

**DOI:** 10.1186/s12951-022-01604-5

**Published:** 2022-08-31

**Authors:** Yang Gao, Yun Li, Hongmei Cao, Haixue Jia, Dianyu Wang, Chunhua Ren, Zhongyan Wang, Cuihong Yang, Jianfeng Liu

**Affiliations:** Key Laboratory of Radiopharmacokinetics for Innovative Drugs, Chinese Academy of Medical Sciences, and Institute of Radiation Medicine, Chinese Academy of Medical Sciences & Peking Union Medical College, Tianjin, 300192 China

**Keywords:** Self-assembled peptide, l-buthionine-sulfoximine (BSO), Glutathione depletion, Glutathione biosynthesis inhibition, Ferroptosis, Pyroptosis

## Abstract

Abundant glutathione (GSH) is a biological characteristic of lots of tumor cells. A growing number of studies are utilizing GSH depletion as an effective adjuvant therapy for tumor. However, due to the compensatory effect of intracellular GSH biosynthesis, GSH is hard to be completely exhausted and the strategy of GSH depletion remains challenging. Herein, we report an l-buthionine-sulfoximine (BSO)-based hypertoxic self-assembled peptide derivative (NSBSO) with dual functions of GSH depletion and biosynthesis inhibition for selective tumor ferroptosis and pyroptosis. The NSBSO consists of a hydrophobic self-assembled peptide motif and a hydrophilic peptide derivative containing BSO that inhibits the synthesis of GSH. NSBSO was cleaved by GSH and thus experienced a morphological transformation from nanoparticles to nanofibers. NSBSO showed GSH-dependent cytotoxicity and depletion of intracellular GSH. In 4T1 cells with medium GSH level, it depleted intracellular GSH and inactivated GSH peroxidase 4 (GPX4) and thus induced efficient ferroptosis. While in B16 cells with high GSH level, it exhausted GSH and triggered indirect increase of intracellular ROS and activation of Caspase 3 and gasdermin E, resulting in severe pyroptosis. These findings demonstrate that GSH depletion- and biosynthesis inhibition-induced ferroptosis and pyroptosis strategy would provide insights in designing GSH-exhausted medicines.

**Graphical Abstract:**

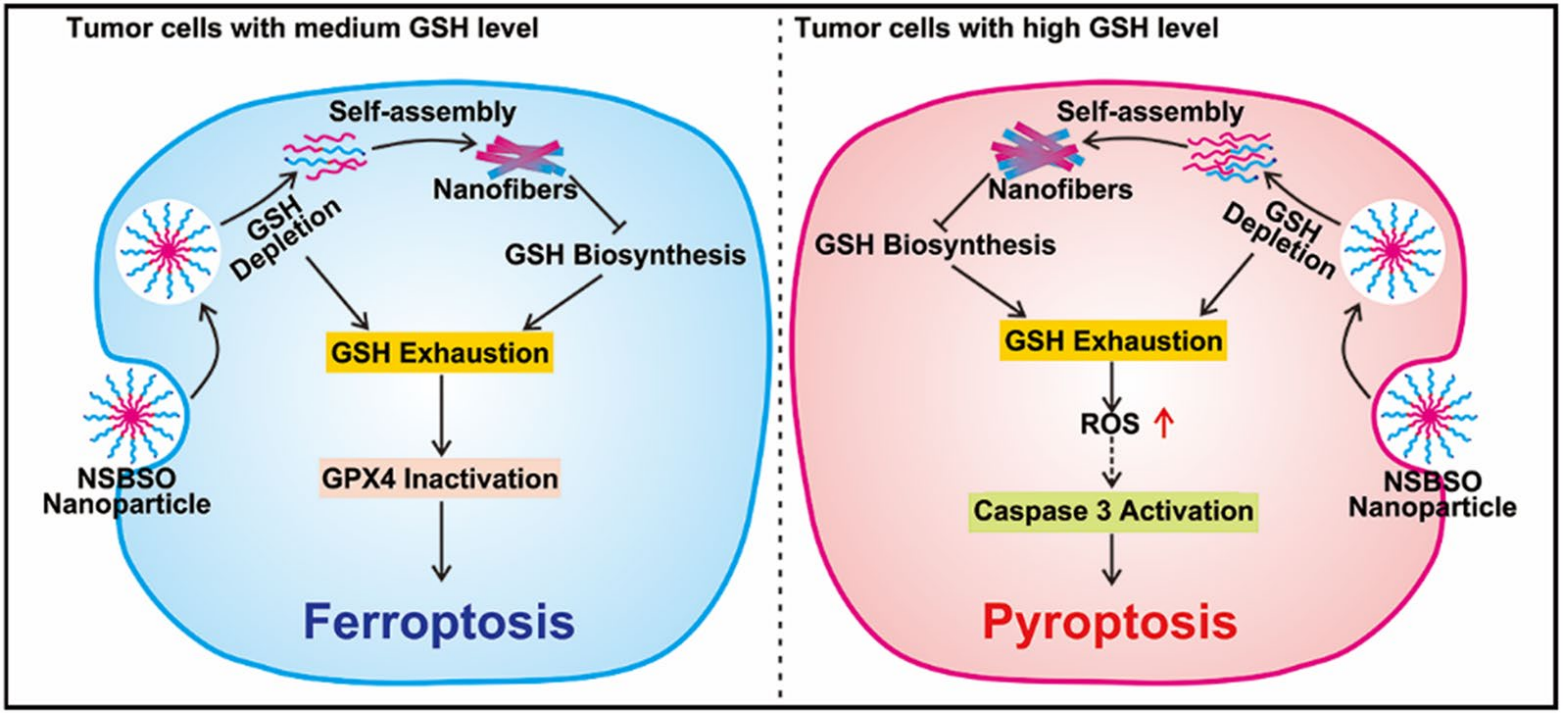

**Supplementary Information:**

The online version contains supplementary material available at 10.1186/s12951-022-01604-5.

## Introduction

Glutathione (GSH) is the most abundant intracellular antioxidant, which plays vital roles in cells, including maintaining redox state, drug detoxification and protecting cells from free radicals, peroxides and toxins. The level of GSH in many kinds of solid tumor tissues was much higher than that in normal tissues and can be over 1000-fold higher than in blood [1, 2]. The elevated GSH level helps to prevent apoptosis of cancer cells by detoxifying reactive oxygen species (ROS) [3] and induce chemotherapeutic drugs resistance through GSH spontaneous reactions or xenobiotics reactions catalyzed by GSH S-transferase (GST) [4, 5]. GSH regulation strategies mainly include the depletion of existing intracellular GSH and the inhibition of GSH biosynthesis. Chemical oxidants can rapidly decrease intracellular GSH, but are easy to produce toxic side effects such as hemolysis and nephrotoxicity [6–9]. Electrophilic reagents such as diethyl maleate (DEM) and isothiocyanates (ITCs) can diminish GSH in the presence of GST, but the high reaction activity of these reagents makes them low targeting and easy to damage normal tissues [10]. Inhibition of glutamate-cysteine ligase (GCL), an important enzyme in GSH biosynthesis, is the most widely used method to inhibit GSH biosynthesis. Among the inhibitors of GCL, l-buthionine sulfoximine (BSO) is the most potent and has already been in the clinical trial stage [11, 12]. What is unsatisfactory is that BSO has no tumor cell selectivity and is easy to metabolize in vivo. Therefore, in the past decade, especially under the promotion of nanotechnology, nanodrugs aimed at depleting GSH or inhibiting GSH biosynthesis have been proposed to improve the effect of tumor treatment and reduce the drug resistance to chemotherapy [10, 13]. However, it is disappointing that with the consumption of intracellular GSH, the cell produces a stress response to up-regulate the biosynthesis of GSH, which will cause intracellular GSH cannot be completely exhausted and cells become resistant to GSH-consuming drugs [14]. Therefore, for the efficient treatment of tumors, the unidirectional regulation of GSH, such as depletion or synthetic inhibition, still faces challenges.

Ferroptosis [15, 16] and pyroptosis [17, 18] are two newly discovered forms of regulatory cell death, which are showing great potential in eradicating invasive malignant tumors resistant to traditional treatments. On the one hand, with the gradual understanding of the mechanism of ferroptosis, researchers have developed a variety of ferroptosis inducers, such as RSL3, FIN56 and BSO [19]. These inducers are mainly small molecule inhibitors. Numerous nanomaterials-based ferroptosis inducers have been reported since the pioneering work in 2016 by Overholtzer and co-workers [20]. However, most of them are metal oxide-based nanoparticles, which suffer from safety concerns when being used in vivo. High levels of GSH inhibit ferroptosis by providing electrons for GSH peroxidases (GPX) to combat lipid peroxidation. Stockwell and co-workers found that depletion of GSH by erastin inactivated GPX enzymes and induced ferroptosis [21]. In addition, most of the nanomaterials that induce ferroptosis by regulating the level of GSH only could be used as auxiliary means of tumor therapy because of the relatively low efficiency of scavenging GSH [11, 22, 23]. On the other hand, ions, small molecule drugs and nanodrugs have been reported to induce tumor cell pyroptosis [17]. Although small-molecule drugs such as metformin and cisplatin are frequently used as pyroptotic inducers, they suffer from rapid clearance, non-specific distribution and systemic side effects. In contrast, nanomaterials can well compensate for these difficulties because of their unique advantages, among which, nanoformulations with tumor microenvironment responsiveness have good prospects for application. Of course, the consideration of the safety of nanomaterials is also indispensable. It has been recently reported that intracellular ROS pathway are widely involved in tumor cell pyroptosis by activating caspases to cut gasdermin family [24, 25]. In view of the balance regulation of GSH and ROS levels in tumor cells, GSH depletion may indirectly induce cell pyroptosis, but it has not been reported yet.

Peptides are widely used in the biomedical field due to their various biological activities, diverse functions, good biocompatibility and biodegradability [26, 27]. As of 2017, 60 peptide drugs have been approved by the United States, Europe and other countries [28]. Recently, peptide self-assembly has become an effective and powerful strategy for the preparation of anti-tumor nanodrugs based on abnormal tumor metabolism and microenvironment, such as overexpression of enzymes [29, 30], acidic pH, high GSH level [31] and specific biomarkers [32, 33]. Among them, the study of peptide self-assembly has entered new and exciting frontiers, including the rational design of peptides or their derivatives to enable them to undergo structural changes or morphological transformation in complex biological environments, especially in cells and in vivo [34]. On the one hand, the rationally designed peptides can achieve rational distribution and overcome the physiological barrier [35]. On the other hand, they can achieve targeted accumulation in interesting sites and reduced toxicity to adjacent normal tissues [36–38].

**Scheme 1:**
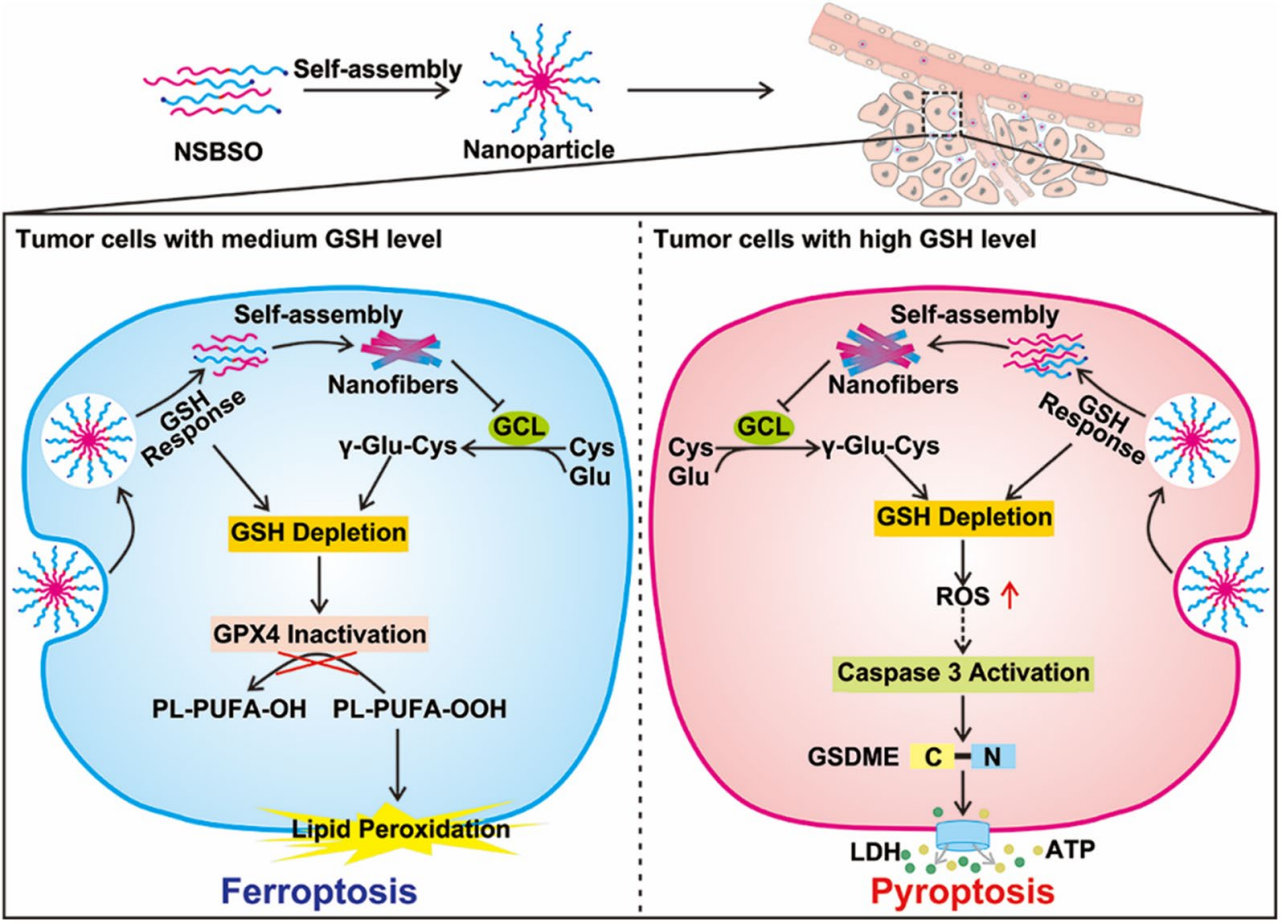
The schematic illustration of GSH-responsive intracellular re-assembly of NSBSO and the selective induction of ferroptosis or pyroptosis in different tumor cells according to the level of intracellular GSH

In this contribution, we constructed a BSO-based hypertoxic self-assembled peptide derivative Nap-^D^F^D^FY-CS-DEVD-BSO (abbreviated as NSBSO) with dual functions of GSH depletion and biosynthesis inhibition for tumor ferroptosis and pyroptosis (Scheme 1). The peptide derivative consists of a hydrophobic self-assembled peptide motif and a hydrophilic peptide motif containing BSO that inhibits the synthesis of GSH. The GSH-responsive intracellular re-assembly from nanoparticles to nanofibers can deplete existing intracellular GSH and achieve minimal effusion and the efficient accumulation of intracellular BSO, which may inhibit the biosynthesis of GSH and aggravate intracellular GSH depletion. Interestingly, NSBSO produces super cytotoxicity through ferroptosis or pyroptosis in different tumor cells, depending on the level of intracellular GSH. Our findings show the effect of depletion of existing intracellular GSH and inhibition of GSH biosynthesis with a BSO-based peptide derivative, which provides a new strategy for in situ construction of selective and efficient nanomedicine for intracellular GSH regulation and tumor inhibition.

## Results and discussion

### Synthesis, in vitro response and transformation of NSBSO to GSH

**Fig. 1:**
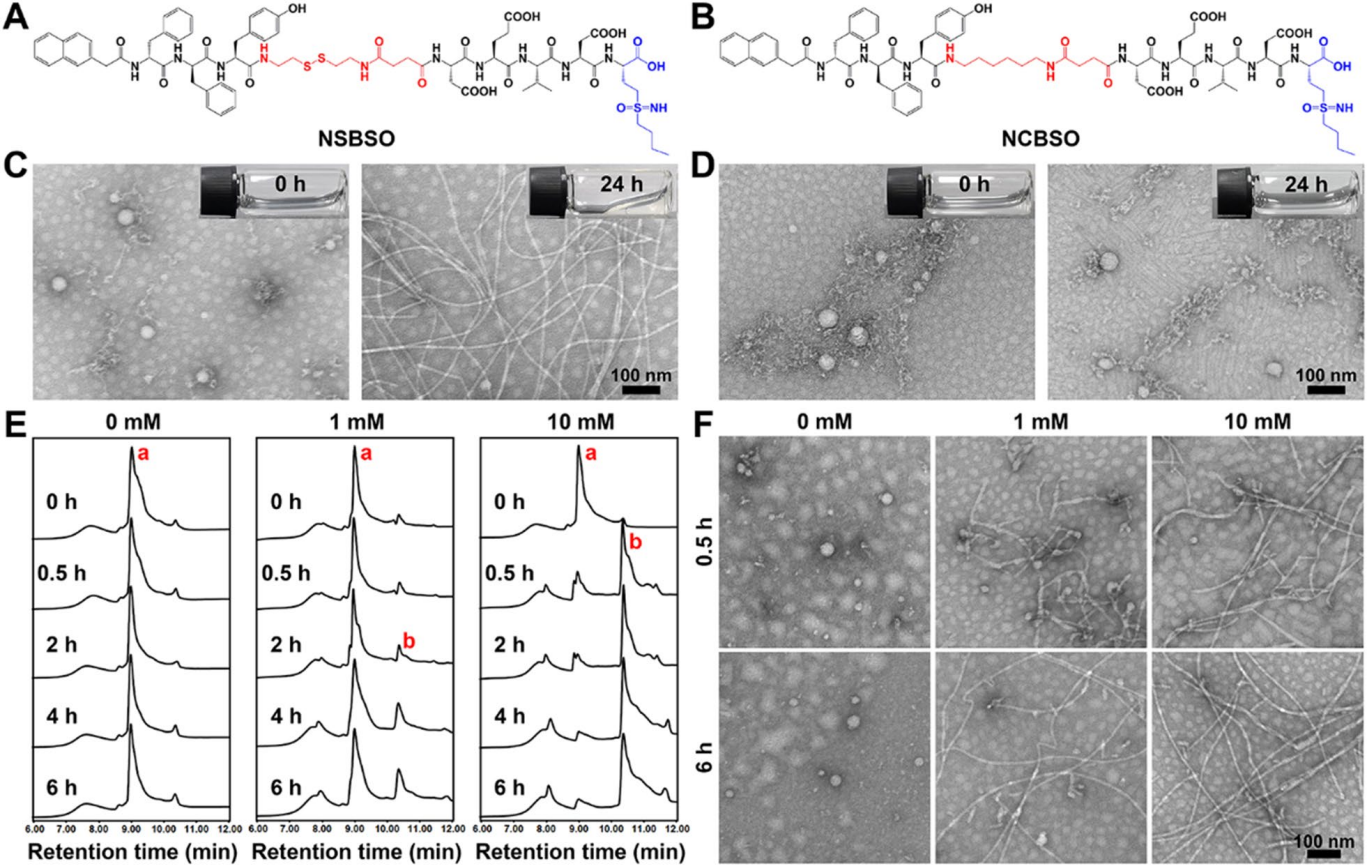
Molecular structure and in vitro response and transformation of NSBSO to GSH. **A**, **B** The molecular structures of NSBSO and NCBSO. **C**, **D** Optical images (inserts) and TEM images of (**C**) NSBSO and (**D**) NCBSO after 10 mM GSH treatment at 37 °C for 24 h. **E** HPLC traces and **F** the corresponding TEM images of NSBSO in the presence of 0 mM, 1 mM and 10 mM GSH at 37 °C within 6 h. The red a and b in (**E**) represents NSBSO and Nap-^D^F^D^FY-thiol, respectively

The designed BSO-based and GSH-responsive peptide derivative (NSBSO) was comprised of the following essential parts (Fig. 1A): (1) Nap-^D^F^D^FY, a hydrophobic peptide capable of self-assembling into nanofibers; (2) DEVD-BSO, the hydrophilic peptide sequence containing BSO to adjust the balance of hydrophilicity and hydrophobicity and inhibit the synthesis of intracellular GSH; (3) succinic anhydride-modified cystamine as a linker of (1) and (2), and had the function of GSH responsive cleavage, which depleted the existing intracellular GSH. Specifically, we first prepared the intermediates, including Fmoc-CS, Fmoc-HDA and Fmoc-BSO, by modifying the disulfide bond linker, the control non-GSH responsive carbon–carbon bond linker and BSO with 9-fluorenylmethyloxycarbonyl (Fmoc) group and succinic anhydride through liquid-phase reactions (Additional file 1: Figs. S1–S3). Then, the peptide derivative NSBSO was obtained by classical solid-phase peptide synthesis using the above intermediates according to our previous literature [39, 40]. All the intermediates and the final product were verified by time-of-flight mass spectrometry (TOF–MS) (Additional file 1: Figs. S4–S7). Meanwhile, to investigate the GSH responsiveness and the function of BSO, the control peptide derivative Nap-^D^F^D^FY-HDA-DEVD-BSO (NCBSO) and peptide derivative without BSO Nap-^D^F^D^FY-CS-DEVD (NS) were prepared respectively in a similar way (Fig. 1B and Additional file 1: Figs. S8, S9). The yields of Fmoc-CS, Fmoc-HDA and Fmoc-BSO were 79.16%, 62.89% and 84.31%, respectively. The yields of NSBSO, NCBSO and NS were 18.18%, 15.69% and 24.7%, respectively.

Transmission electron microscopy (TEM) results showed that NSBSO and NCBSO spontaneously assembled into spherical nanoparticles (NPs) after dissolving in phosphate buffered saline (PBS) solution with the concentration of 1 mM, with the average diameter of 48.26 ± 6.17 nm and 41.27 ± 6.62 nm, respectively (Fig. 1C, D). These results confirmed that NSBSO and NCBSO had similar self-assembling properties. The GSH responsiveness of NSBSO and NCBSO were validated by incubating them with GSH (10 mM) for 24 h. As shown in Fig. 1C inserts, the transparent solution of NSBSO turned into sticky hydrogel after the treatment of GSH for 24 h, but not NCBSO (Fig. 1D inserts). The TEM results were consistent with the visual changes, which illustrated the morphological transformation of NSBSO from nanoparticles to regular nanofibers with diameters of ~ 10 nm and the lengths of micrometers (Fig. 1C). While NCBSO nanoparticles were stable in aqueous solution after GSH treatment (Fig. 1D).

We further investigated the molecular changes of NSBSO after GSH treatment by high-performance liquid chromatography (HPLC) analysis. When NSBSO is reduced by GSH, the disulfide bond of NSBSO will break and NSBSO will then be converted to Nap-^D^F^D^FY-thiol and this process can be detected by liquid chromatography-mass spectrometry (LC–MS) analysis (Additional file 1: Fig. S10). As shown in Fig. 1E and Additional file 1: Fig. S11, NSBSO was extremely stable in PBS buffer solution without GSH and conversion from NSBSO (a) to Nap-^D^F^D^FY-thiol (b) occurred after GSH treatment. Notably, the reduction of NSBSO showed a significant dependence on the time and concentration of GSH treatment. Specifically, in the presence of 1 mM GSH, NSBSO gradually converted to Nap-^D^F^D^FY-thiol, and the conversion rate was close to 25% after treatment for 6 h. In comparison, when the concentration of GSH was increased to 10 mM, 74% of NSBSO converted to Nap-^D^F^D^FY-thiol within 0.5 h and the conversion rate was as high as 92% after 4 h treatment. As revealed in Fig. 1F, the results of TEM images showed the morphological transformation of NSBSO both in the presence of 1 mM and 10 mM GSH, which was highly consistent with the results of HPLC traces. It can be found that nanofibers have begun to form after treatment of 1 mM GSH for 0.5 h, which may be related to Nap-FFY as an effective supramolecular gelator [41, 42]. These above results indicate that NSBSO is highly sensitive to GSH treatment and is closely related to the time and concentration of GSH treatment. Moreover, the results indicate that morphological transformation of NSBSO solution from nanoparticles to nanofibers is realized after GSH treatment. The stability of NSBSO in 10% serum solution was analyzed. The HPLC results showed that the stability of NSBSO was more than 80% after incubation for 2 h, and more than 50% for 24 h (Additional file 1: Fig. S12). This may be because the self-assembled peptides usually have better stability after forming nanostructures than their corresponding small molecules, and the core assembly sequence of NSBSO was composed of two d-phenylalanines, which can better resist the degradation of enzymes than l-counterparts. We further studied the blood cell compatibility of NSBSO by hemolysis experiment. The results in Additional file 1: Fig. S13 showed that NCBSO, NS and NSBSO did not cause hemolysis in the concentration range of 62.5–4000 μM, indicating that all materials had good ability to coexist with the membrane.

### GSH-dependent cytotoxicity and depletion of intracellular GSH

**Fig. 2:**
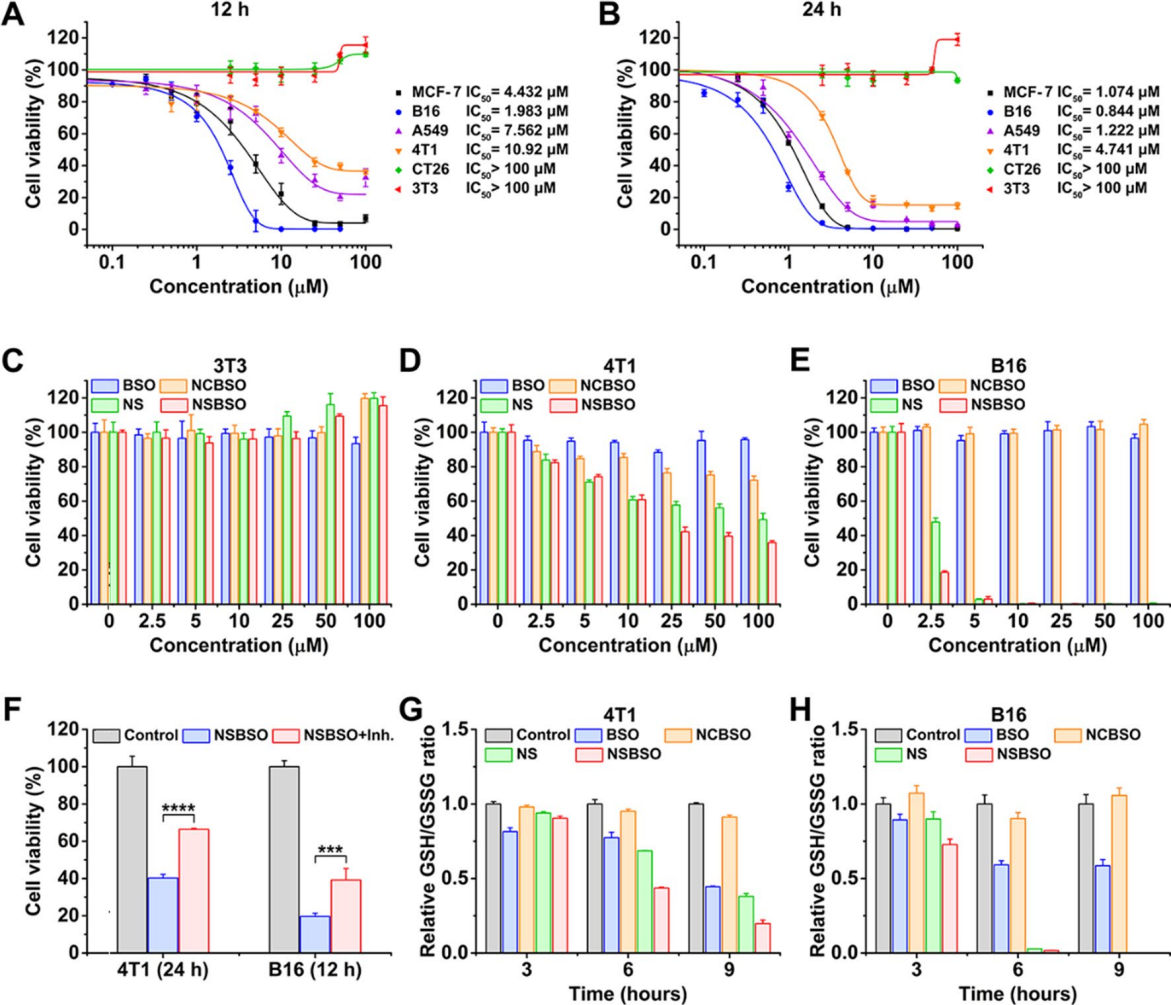
GSH-dependent cytotoxicity and depletion of intracellular GSH. **A**, **B** Cytotoxicity and IC_50_ values of NSBSO against different cells after incubation for 12 h and 24 h. **C**–**E** Cytotoxicity of NSBSO and other formulations against 3T3 cells, 4T1 cells and B16 cells after incubation for 12 h. **F** The cell viability of NSBSO in 4T1 and B16 cells w or w/o pre-treatment of GCL inhibitor (Data are presented as mean ± SD, *n* = 4; ***P < 0.001 and ****P < 0.0001). **G**, **H** GSH/GSSG level in 4T1 cells and B16 cells treated with 10 μM formulations for different duration (Data are presented as mean ± SD, *n* = 3)

After validating that NSBSO is highly sensitive to GSH treatment in vitro, we explored its cellular effect induced by this molecular and morphological transformation and the simultaneous GSH regulation by measuring the cell viability in cck-8 assay. As shown in Fig. 2A and B, the calculated half-maximal inhibitory concentration (IC_50_) values were completely different for different cell lines. When the concentration of NSBSO was as high as 100 μM, it had no obvious cytotoxicity to CT26 and NIH3T3 cells. But, notably, 24 h treatment of NSBSO showed strong cytotoxicity against MCF-7, B16, A549 and 4T1 cells, with IC_50_ values of 1.074, 0.844, 1.222 and 4.741 μM, respectively, which were much lower than those of reported GSH-responsive peptide derivatives whose IC_50_ was greater than 100 μM [43]. What’s more, the IC_50_ values of peptide derivatives covalently linked to chemotherapeutic agents (e.g. curcumin, podophyllotoxin) were still in the micromolar range [44, 45].

To determine whether the difference of IC_50_ values is related to the cellular GSH level, we examined the intracellular GSH level using the GSH/GSSG detection kit. As shown in Additional file 1: Fig. S14, tumor cells showed relatively higher GSH than normal cells (3T3 cells) and B16 cells showed the highest intracellular GSH level, which were consistent with previous reports [1, 2]. Specifically, the intracellular GSH levels from high to low displayed as B16 > MCF-7 > A549 > 4T1 > CT26 > 3T3, which had a positive correlation with the cytotoxicity of NSBSO on these cell lines (Fig. 2A, B), indicating that the cytotoxicity of NSBSO is caused by the intracellular GSH response.

Then, we selected B16, 4T1 and 3T3 cells as representatives of relatively high, medium and low GSH levels of cells for in-depth study. Cells were treated with NSBSO and control materials (BSO, NS and NCBSO) for 12 h and 24 h and cck-8 assay was performed. As shown in Fig. 2C and Additional file 1: Fig. S15, all formulations had no obvious cytotoxicity to 3T3 cells within the given concentration range. We found that NS and NSBSO showed concentration-dependent cytotoxicity in both 4T1 cells and B16 cells, which should be due to the relatively high level of GSH in 4T1 cells and B16 cells (Fig. 2D, E and Additional file 1: Figs. S16, S17). Moreover, the GSH level of B16 cells with a stronger killing effect was 2.1 times higher than that of 4T1 cells (Additional file 1: Fig. S14). However, NCBSO and free BSO, which did not have GSH-responsive properties, did not show obvious cytotoxicity within the given concentration range. In addition, although NS had selective cytotoxicity similar to that of NSBSO, its toxicity was lower. These differences showed that both the disulfide bond and BSO in NSBSO play an important role in killing cells.

Subsequently, to verify the effect of different GSH levels on NSBSO toxicity in the same cell lines, we pretreated 4T1 and CT26 cells with different concentrations of GSH before NSBSO treatment. The results showed that artificially increase the intracelluar GSH level could enhance the cytotoxicity of NSBSO (Additional file 1: Fig. S18). To further prove the influence of intracellular GSH level on the cytotoxicity of NSBSO, we pre-treated 4T1 and B16 cells with GSH synthesis inhibitor to reduce the intracellular GSH level, and the results showed that the pretreatment of the inhibitors gradually reduced the intracellular GSH to a very low level after 24 h treatment (Additional file 1: Fig. S19). But the cell morphology at 24 h after GCL inhibitor treatment did not change in both cells (Additional file 1: Fig. S20), which was consistent with the results reported in the literature [46]. As a result, the addition of inhibitor remarkably compromised the cytotoxicity of NSBSO (Fig. 2F), demonstrating the cytotoxicity of NSBSO was closely related to the high level of intracellular GSH.

**Fig. 3:**
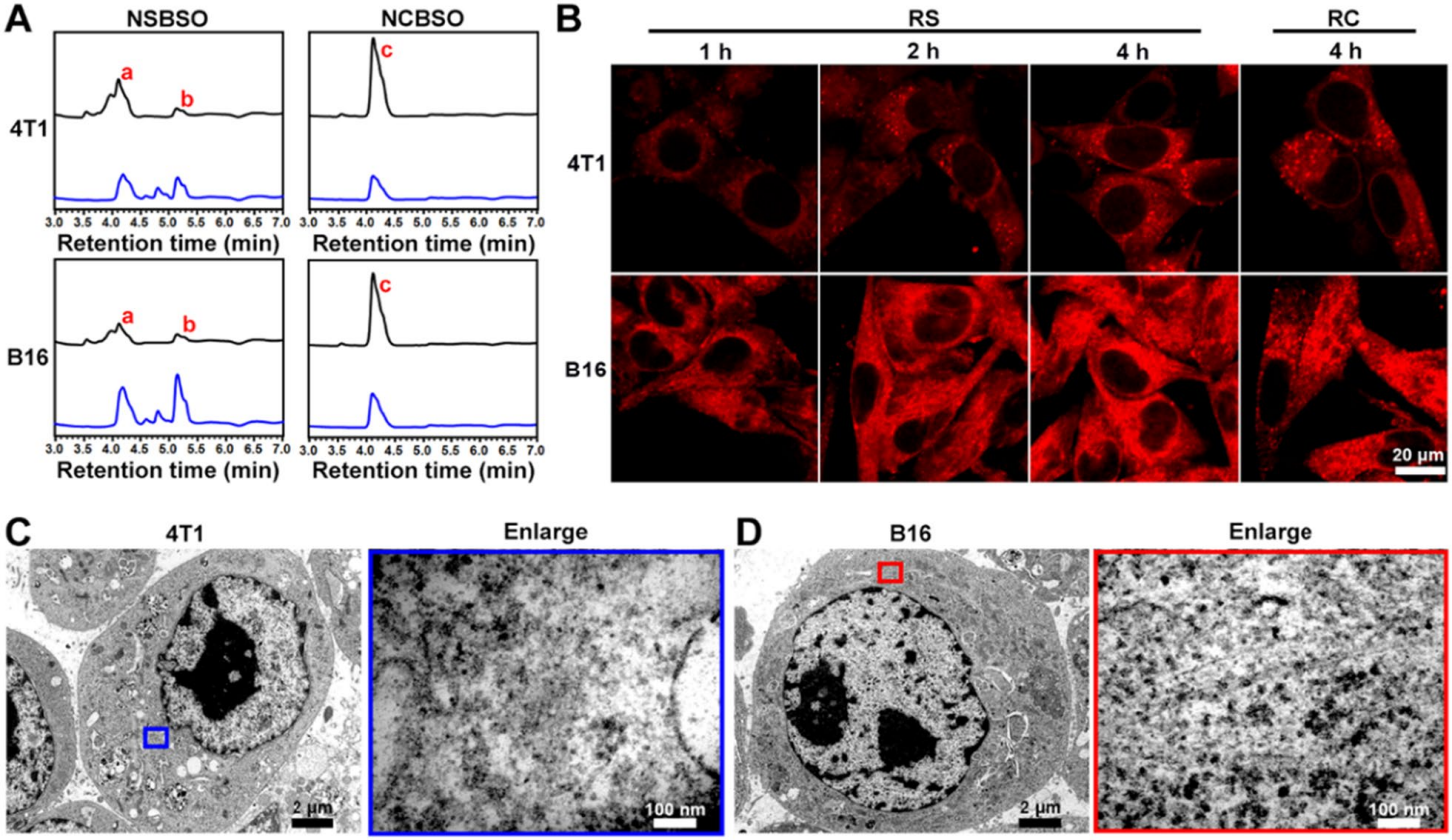
Intracellular GSH-responsive transformation. **A** LC–MS traces of the cellular uptake and transformation of 100 μM NSBSO and NCBSO after incubation with 4T1 and B16 cells for 10 h (black lines indicate culture medium, blue lines indicate cell lysates). **B** CLSM images of 4T1 and B16 cells treated with RS or RC (100 μM). **C**, **D** Bio-TEM image of 4T1 cell and corresponding higher-magnification image of the blue-framed area, and B16 cell and corresponding higher-magnification image of the red-framed area after 4-h treatment of NSBSO treatment (100 μM for 4T1 cells, 10 μM for B16 cells). The red a, b and c in (**A**) represents NSBSO, Nap-^D^F^D^FY-thiol and NCBSO, respectively

In addition, we evaluated the intracellular GSH level after different treatments. As revealed in Fig. 2G and H, after treatment of NSBSO, the GSH/GSSG level in 4T1 cells decreased rapidly with the increase of incubation time. What is more noteworthy was that the GSH/GSSG level of B16 cells decreased rapidly and decreased to about 0 at 6 h, which may be due to the GSH-responsive transformation of NSBSO in B16 cells (will be illustrated in Fig. 3). NSBSO rapidly responded in high GSH B16 cells and consumed part of GSH, and then the resultant Nap-^D^F^D^FY-thiol and NSBSO co-assembled into nanofibers in the cell, allowing more BSO to accumulate, thereby inhibiting intracellular GSH synthesis and ultimately minimizing intracellular GSH. The level of GSH/GSSG in the cells treated with NS for more than 6 h was also significantly decreased, but the extent was smaller than that in the NSBSO group, indicating that the combination of consumption of existing GSH and inhibition of GSH synthesis promotes the exhaustion of GSH in cells. Furthermore, the intracellular ROS level was detected by ROS probes. It was found that the intracellular ROS level of 4T1 and B16 cells also increased significantly after treatment with NSBSO or NS, and more ROS was produced in B16 cells than in 4T1 cells (Additional file 1: Fig. S21). These results indicate that NSBSO has GSH-dependent cytotoxicity, which may be related to the efficient depletion of GSH.

### Intracellular GSH-responsive transformation

The in situ assembly and morphology transformation of peptide derivatives in tumor cells have greatly promoted the treatment of tumors. In order to investigate the intracellular molecular transformation and the morphology transformation of NSBSO which showed good GSH-response in vitro and excellent selective cellular toxicity, we conducted LC–MS, confocal laser scanning microscopy (CLSM) and Bio-TEM analysis. As shown in Fig. 3A and Additional file 1: Figs. S22–25, after co-incubation with 4T1 and B16 cells for 10 h, NSBSO (a) converted to Nap-^D^F^D^FY-thiol (b) in both cell lines, while NCBSO (c) did not undergo intracellular conversion. Due to the presence of small amount of GSH outside the tumor cells [47], NSBSO had also undergone a certain degree of extracellular conversion. It was worth noting that the conversion ratio of NSBSO in B16 cells was higher than that in 4T1 cells (50% and 40%, respectively) (Additional file 1: Figs. S24, S25). We speculated that this may be related to the higher level of GSH in B16 cells compared to 4T1 cells [48]. To further observe the transformation of peptides in living cells, we conjugated a fluorescent indicator Rhodamine B (RhoB) to peptides and synthesized RS and RC as analogs of NSBSO and NCBSO respectively (Additional file 1: Figs. S26, S27) and performed the cellular uptake experiment in 4T1 and B16 cells. As shown in Fig. 3B, the fluorescence intensity of RS increased with time both in 4T1 and B16 cells. Notably, RS showed scattered fluorescent dots inside 4T1 cells, while obvious filamentous aggregation in B16 cells. By comparison, although the intracellular accumulation of RC in both cells increased over time (Additional file 1: Fig. S28), its fluorescence intensity was much lower than that treated with RS, but it was less than that of RS, and most of the intracellular RC showed punctate distribution, which was significantly different from that of RS in B16 cells. To directly investigate the intracellular formation of molecular assemblies, we utilized Bio-TEM to image the cells. It can be seen from Fig. 3C and D that NSBSO formed nanofibrous aggregates in cell plasma, especially in the cytoplasm of B16 cells. These results indicated that RS could respond to intracellular GSH and transform into fibrous nanostructure in high GSH level B16 cells, which is consistent with the results of LC–MS traces (Fig. 3A) and the rapid depletion of GSH in B16 cells after NSBSO treatment (Fig. 2H). Moreover, the intracellular transformation of NSBSO and the formation of nanofibers may be related to its high cytotoxicity.

### GSH-regulated tumor cell ferroptosis and mechanism study

**Fig. 4:**
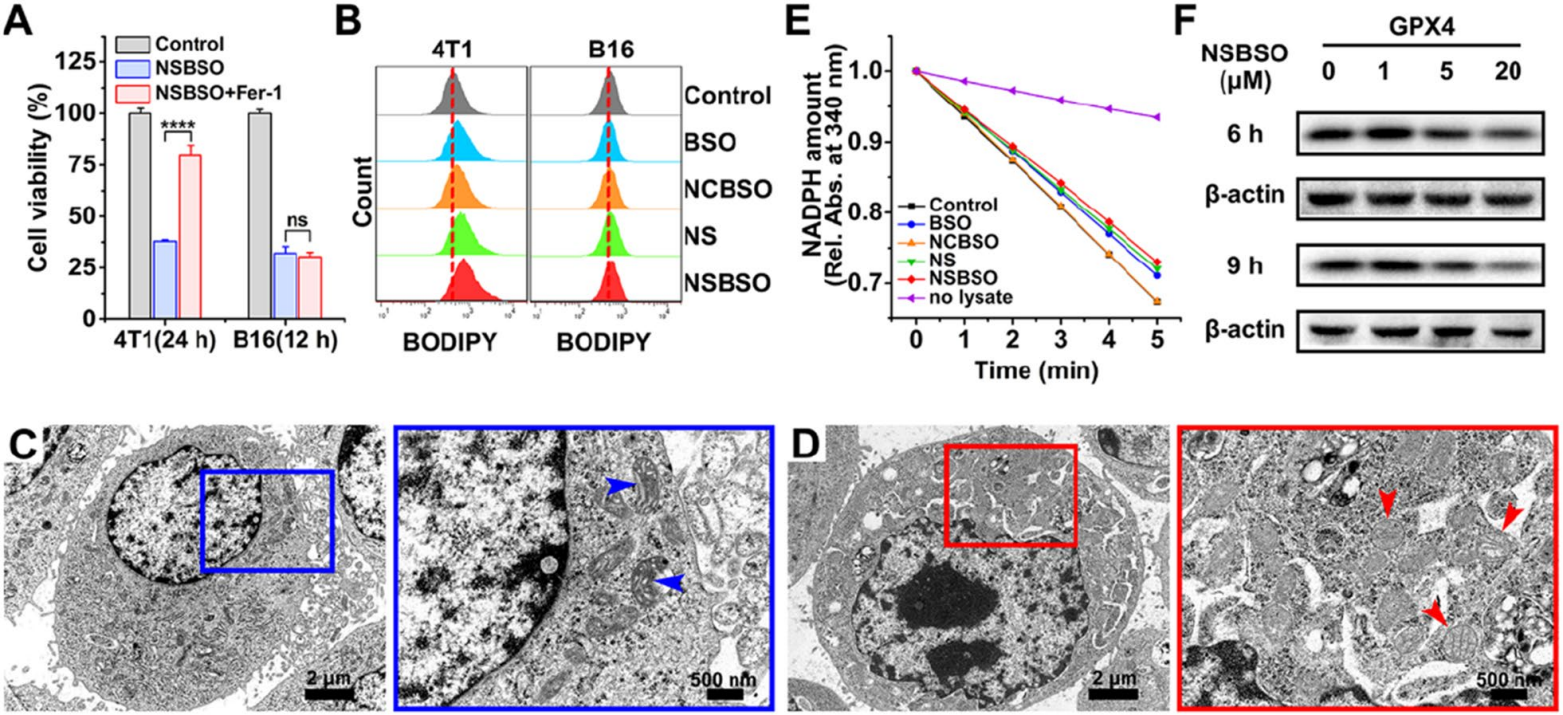
Ferroptosis of 4T1 cells induced by NSBSO and the mechanism study. **A** The cell viability of 4T1 and B16 cells treated with NSBSO in the presence or absence of ferroptosis inhibitor (Fer-1, 0.5 μM) (Data are presented as mean ± SD, *n* = 4; ****P < 0.0001). **B** Flow cytometry analysis of BODIPY dye-stained 4T1 and B16 cells treated with different formulations (10 μM, 10 h). **C**, **D** Bio-TEM images of (**C**) an untreated 4T1 cell and (**D**) NSBSO treated 4T1 cell, and corresponding higher-magnification images of the blue and red boxes (arrowheads indicate mitochondria). **E** The GPX activity of 4T1 cells measured by the relative absorbance of NADPH reduction at 340 nm (10 μM, 6 h). **F** The expression level of GPX4 in 4T1 cells treated with different times and different concentrations of NSBSO

Based on the above research results, NSBSO showed a very strong selective killing ability of tumor cells by exhausting GSH and inhibiting GSH synthesis. Therefore, we next studied the specific mechanism of tumor cell death caused by NSBSO. It has been reported that GSH depletion or the inactivation of glutathione peroxidase 4 (GPX4) can induce ferroptosis [21, 49, 50]. In order to verify whether ferroptosis occurred in 4T1 cells and B16 cells, we pretreated cells with ferrostatin-1 (Fer-1, 0.5 μM), a ferroptosis inhibitor that prevents the formation of lipid peroxides via a reductive mechanism [51], 24 h before NSBSO treatment. As shown in Fig. 4A and Additional file 1: Figs. S29 and S30, for 4T1 cells, treatment with Fer-1 rescued NSBSO-induced cell death by 42.13%, whereas treatment with Fer-1 did not markedly alleviate the NSBSO-induced B16 cells death. Besides, Z-VAD-FMK, an apoptosis inhibitor, scarcely rescued NSBSO-induced cell death in 4T1 cells (Additional file 1: Fig. S31). These results indicated that ferroptosis might play an important role in NSBSO-induced 4T1 cells death, while B16 cells may have different death patterns.

To further verify the mechanism of NSBSO-induced cell death in 4T1 cells, lipid peroxidation and micromorphology of mitochondria were examined. We first used BODIPY^581/591^ C11, a lipid peroxidation probe, to detect the lipid peroxide level of NSBSO treated 4T1 cells. As displayed in Fig. 4B and Additional file 1: Fig. S32, the level of lipid peroxidation in 4T1 cells increased by 8.2% and 14% after NS and NSBSO treatment, respectively. However, there was no significant change in B16 cells after different treatments. We then monitored the micromorphological changes of 4T1 cells by Bio-TEM imaging. Compared with untreated cells (Fig. 4C), NSBSO treated 4T1 cells exhibited shrunken mitochondria with decreased mitochondria cristae, morphological changes from ellipsoid to spheroid, accompanied with damage of mitochondria (Fig. 4D). These micromorphological changes of mitochondria were highly consistent with the characteristics of ferroptosis [15].

In addition, the activity and expression level of GPX4, which plays the role of eliminating lipid peroxides and inhibiting ferroptosis, were studied. The reducing activity of GPX4 in cell lysates was examined by monitoring the rate of NADPH oxidations using tert-butylhydroperoxide (t-BuOOH) as the substrate. As can be seen in Fig. 4E, as the oxidation rate of NADPH (shown by the decrease of absorption in 340 nm) was comparable with the control group, NCBSO treatment did not decrease the activity of GPX4. But the activity of GPX4 was all suppressed to varying degrees in cells treated with BSO, NS and NSBSO, respectively. Notably, NSBSO treatment showed the strongest inhibition of GPX4 reducing activity. The results of protein expression in western blot also showed that NSBSO treatment for 9 h significantly decreased the expression of GPX4 (Fig. 4F). The GCL level was always detected by western blot. As show in Additional file 1: Fig. S33, the expression of GCL decreased in a concentration-dependent manner after NSBSO treatment for 9 h. These results indicated the occurrence of ferroptosis in NSBSO treated 4T1 cells. In addition, for NSBSO treated B16 cells, the expression of GPX4 did not have obvious change, but the expression of GCL decreased obviously (Additional file 1: Figs. S34, S35), further suggesting that NSBSO could induce ferroptosis in 4T1 cells through GPX4 inhibition.

Taken together, these findings indicate that NSBSO treatment leads to GSH depletion and inhibition of GSH biosynthesis, which in turn inhibits GPX4 activity and resulting ferroptosis in 4T1 cells. Meanwhile, the depletion of intracellular GSH is accompanied by the in situ co-assembly of NSBSO and NSBSO reduction products, which can inhibit the biosynthesis of GSH more efficiently than free BSO which is easily metabolized from cells.

### GSH-regulated tumor cell pyroptosis and mechanism study

Since Fer-1 (a ferroptosis inhibitor) failed to alleviate the cytotoxicity of NSBSO against B16 cells, we further investigated the mechanism of the rapid and intense death of B16 cells induced by NSBSO. We noted that intracellular oxidative stress detected by 2,7-dichlorodihydrofluorescein diacetate (DCFH-DA) of B16 cells treated with NSBSO was 2.24-fold higher than that of 4T1 cells (Additional file 1: Fig. S21), which was consistent with the results that B16 cells showed more significant GSH depletion than 4T1 cells (Fig. 2G, H). Intracellular ROS has been recently proposed to involve in pyroptosis in tumor cells [24, 25]. However, the specific mechanisms by which ROS participates in pyroptosis are complex and still unclear. We hypothesized that NSBSO-induced GSH depletion triggers pyroptotic cell death in B16 cells. To investigate this, we carried out cell morphology observation, lactate dehydrogenase (LDH)-release test, protein level study and Annexin V-FITC/PI double staining analysis.

**Fig. 5:**
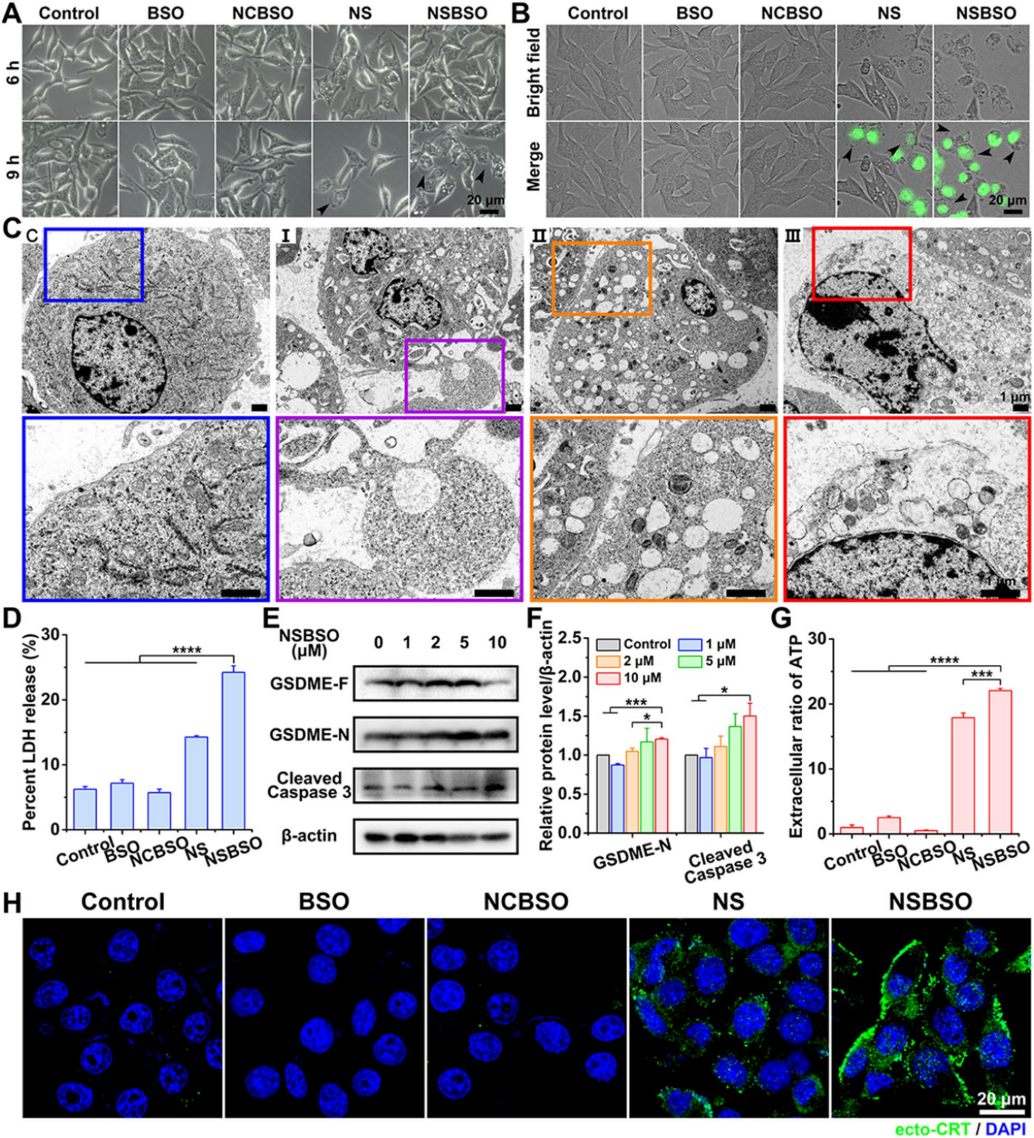
Pyroptosis of B16 cells induced by NSBSO and the mechanism study. **A** Representative images of the morphology of B16 cells treated with different formulations (10 μM). **B** Fluorescence images of B16 cells treated with different formulations (10 μM) for 10 h (SYTOX green stained nuclei of dying cells, black arrows indicate cell swelling with big bubbles). **C** Bio-TEM images of B16 cells at different stages after 10 μM NSBSO treatment and corresponding higher-magnification images of the boxes. **D** LDH release of B16 cells treated with different formulations for 9 h (Data are presented as mean ± SD, *n* = 3; ****P < 0.0001). **E**, **F** The expression level (**E**) and semi-quantification analysis (**F**) of GSDME-N and Cleaved Caspase 3 in B16 cells treated with different concentrations of NSBSO for 6 h (Data are presented as mean ± SD, *n* = 3; *P < 0.05 and ***P < 0.001).** G** Extracellular ratio of ATP of B16 cells treated with different formulations for 9 h (Data are presented as mean ± SD, *n* = 3; ***P < 0.001 and ****P < 0.0001). **H** Immunofluorescence images of ecto-CRT in B16 cells treated with different formulations (10 μM) for 10 h

Firstly, careful inspection of the morphology of B16 cells with various treatments was carried out by optical and fluorescence microscopy. As displayed in Fig. 5A, compared with the control group of non-treated, BSO-treated and NCBSO-treated cells, bright spots gradually appeared on the cell surface after 6 h of NS and NSBSO treatment, and extensive bright spots were observed in B16 cells treated with NSBSO for 9 h. After incubation for about 10 h, a large number of B16 cells showed SYTOX green staining, which is a nuclei dye of dead cells. Meanwhile, most B16 cells showed obviously pyroptotic morphology, with swelling and characteristic large bubbles from the plasma membrane (Fig. 5B), indicating that pyroptosis occurred in NSBSO-treated B16 cells. By contrast, no similar pyroptotic morphology was observed in 4T1 cells treated with different formulations (Additional file 1: Fig. S36). The fine morphological changes of B16 cells treated with NSBSO for 6 h to 10 h were further examined via Bio-TEM, so as to verify the pyroptosis process of B16 cells. For untreated control cells, the cell membrane was intact (Fig. 5C(C)). For NSBSO-treated cells, we found pore formation on cell membranes and large bubbles blowing from the plasma membrane (Fig. 5C(I)). We also observed the rupture of bubbles (Fig. 5C(II)) and the partial and complete destruction of the plasma membrane (Fig. 5C (III)). Then, the LDH release, as a key indicator of cell pyroptosis was conducted. As shown in Fig. 5D, 3.9-, 3.4- to 4.2-fold increase of LDH release (versus control, BSO and NCBSO, respectively) were observed in cells treated with NSBSO, further validating the occurrence of pyroptosis.

We then sought to investigate the mechanism of pyroptosis in NSBSO-treated B16 cells. Gasdermin D (GSDMD) and gasdermin E (GSDME), cleaved by Caspase 1 and Caspase 3 respectively, are two main discovered executors of pyroptosis [17, 52]. We found that Ac-DEVD-CHO, a caspase 3 inhibitor, better rescued cytotoxicity of NSBSO against B16 cells when comparing with the caspase 1 inhibitor (Z-YVAD-FMK) (Additional file 1: Fig. S37). Moreover, we detected increased expression of cleaved caspase 3 and decreased GSDME cleavage product (GSDME-N) in NSBSO-treated cells (Fig. 5E, F). Meanwhile, there was no great difference between the expression of Caspase 1 and GSDMD with the increase of incubation concentration in NSBSO treated B16 cells (Additional file 1: Fig. S38). To verify the key role of GSH response in inducing pyroptosis, we compared the expression of GSDMD and GSDME in 4T1, B16 and CT26 cells. We found that although CT26 cells had high levels of GSDMD and GSDME expression (Additional file 1: Fig. S39), NSBSO treatment did not induce cell pyroptosis because of its low intracellular GSH level (Fig. 2A, B and Additional file 1: Fig. S14). In order to further prove the role of Caspase 3 in inducing pyroptosis, we performed Annexin V-FITC/PI double staining analysis to distinguish pyroptosis and apoptosis according to literature reports [52–54]. The results in Additional file 1: Fig. S40 indicated that significant pyroptosis occurred in B16 cells after NSBSO treatment. Moreover, there was no obvious early apoptotic cells, which basically ruled out the occurrence of apoptosis.

Pyroptosis is generally considered as a form of immunogenic cell death (ICD). We detected the extracellular release of adenosine triphosphate (ATP) from B16 cells treated with NSBSO and the results in Fig. 5G showed that remarkable extracellular ATP release was found after NSBSO treatment for 9 h. In addition, surface-exposed calreticulin (ecto-CRT) is also one of many molecular events related to ICD. We detected the CRT exposure of B16 cells treated with NSBSO by immunofluorescence. Compared with blank control, BSO and NCBSO groups, a small amount of fluorescence appeared after 10 h of NS treatment, while NCBSO group showed a time-dependent increase of fluosescence within 10 h (Additional file 1: Fig. S41), and a large amount of fluorescence appeared on the cell membrane at 10 h (Fig. 5H), which indicated a large amount of exposure of CRT and the occurrence of ICD. These results suggest that NSBSO-induced pyroptotic cell death in B16 cells is mediated by GSDME activation. Taken together, these findings suggest that NSBSO-induced GSH depletion and synthesis inhibition can elevate intracellular ROS, thereby activating the caspase family, leading to GSDME cleavage and cell pyroptosis.

## Conclusion

In summary, we constructed a BSO-based hypertoxic self-assembled peptide derivative. It self-assembled into spherical nanoparticles and realized the morphology transformation from nanoparticles to nanofibers in the presence of GSH, showing excellent GSH-responsive property in vitro. In addition, it showed GSH-dependent high cytotoxicity on account of the rapid exhaust of intracellular GSH by both GSH depletion and biosynthesis inhibition. Importantly, we found that in 4T1 cells with moderate GSH level, it induced ferroptosis by consuming intracellular GSH and in turn inactivating GPX4. While in B16 cells with high GSH level, it indirectly increased the level of intracellular ROS by GSH depletion and activated Caspase 3 and then GSDME to induce pyroptosis. Our research provides a new perspective for the development of safer and more efficient anticancer nanomedicine for tumor ferroptosis and pyroptosis. We believe that the GSH-regulation strategy of simultaneously depleting GSH and inhibiting GSH synthesis by BSO-based self-assembled peptide derivative would provide inspiration for the design of GSH-exhausted nanomedicines.

## Methods

### Self-assembly and GSH response of peptide derivatives

1.54 mg of NSBSO was dispersed in 1 mL of PBS and Na_2_CO_3_ (4 equiv to NSBSO) was added subsequently to adjust the pH value to 7.4. Then the clear solution of NSBSO (1 mM) was obtained. After that, GSH (10 mM) was added to the solution of NSBSO with incubation at 37 °C for 24 h to trigger the GSH response, resulting in poor fluidity of the solution. The solution of NSBSO with or without GSH treatment was then analyzed by LC–MS. The other solution of NCBSO was prepared by a similar procedure. The fluidity of NCBSO solution was still good after incubating with GSH for 24 h.

### GSH-responsive transformation

The solution of NSBSO (100 μM) for this experiment was prepared using the same method as described above. Different amounts of GSH were added to the solution, individually, to a final GSH concentration of 0, 1 and 10 mM. All samples were incubated at 37 °C. Each sample (100 μL) was taken out and mixed with acetonitrile (100 μL) to terminate the reaction at predefined time points (0.5, 2, 4 and 6 h). All samples were then centrifuged and tested by HPLC.

### In vitro cell cytotoxicity study

All cells (8 × 10^3^ per well) were plated in 96-well plates at 37 °C and 5% CO_2_ overnight. Then cells were treated with gradient concentrations of NSBSO, NCSBSO, NS or BSO for another 12 h or 24 h. Afterward, cck-8 reagent was added to each well. And the 96-well plates were incubated for another 3 h at 37 °C and followed by determining the absorbance at 450 nm utilizing a microplate reader (Thermo, USA). IC_50_ values of NSBSO against different cells were conducted using GraphPad Prism 8 software. Additionally, in order to determine the cell cytotoxicity of NSBSO after treatment with various inhibitors or GSH, the cells were pretreated with inhibitors for 24 h or GSH (0, 1, 2.5 and 5 mM) for 12 h before NSBSO treatment.

### Intracellular GSH content detection assay

The intracellular GSH content of different kinds of cells and the GSH/GSSG ratio in cells treated with different formulations were both monitored by the same method. Cells were all inoculated in 6-well plates at a density of 4 × 10^5^ cells per well. For measuring intracellular GSH content of different kinds of cells, cells were collected after 24 h with rinsed twice by PBS and the cell numbers were calculated using a cell counter. As for the measurement of GSH/GSSG ratio, plate-adhered 4T1 and B16 cells were incubated with BSO, NCBSO, NS and NSBSO for different times (3, 6, and 9 h) with the same concentration (10 μM). Then cells were collected and washed with PBS twice. At last, the GSH content and GSH/GSSG ratio were detected using a GSH and GSSG assay kit according to the manufacturer’s instructions.

### Cellular uptake

We compared the cellular uptake of NSBSO and NCBSO by LC–MS trace and CLSM imaging. Six-well plate-adhered 4T1 and B16 cells were treated with culture media containing NSBSO and NCBSO of the same concentration (100 µM) at 37 °C for 10 h. Subsequently, the cell culture supernatants and cells in each well were collected separately, and then mixed with 500 µL of DMSO. Thereafter, the mixtures were all centrifuged and the supernatants were analyzed by LC–MS. Furthermore, 4T1 and B16 cells seeded into confocal dishes with a cell density of 2 × 10^5^ cells per dish for 24 h were co-incubated with fresh media containing RS or RC (100 μM). At the indicated time, the culture media were discarded followed by wash three times with PBS and fixation with 4% paraformaldehyde. After that, the cells were imaged by CLSM (Nikon, C2, Japan).

### Intracellular ROS and lipid peroxide measurement

4T1 and B16 cells were seeded into six-well plates in 3 × 10^5^ cells per well and incubated for 24 h to allow the attachment of cells. After being co-incubated with various formulations for predetermined time at 37 °C, the cell culture medium was washed away with PBS. For ROS measurement, DCFH-DA (10 µM) was added with further incubation of 30 min. For lipid peroxide evaluation, cells were incubated for another 30 min after being stained with BODIPY^581/591^ C11 probe (2.5 μM). Thereafter, all cells were collected for quantitative analysis by flow cytometry (Invitrogen, Attune NxT, USA) and FlowJo 7.6.1.

### Intracellular GPX4 activity assay

4T1 cells were all seeded in the density of 1 × 10^6^ cells per dish in 6-cm dishes for 24 h. Upon completion of incubation with different treatments for 6 h, the cell lysates were collected and quantified by a detergent compatible bradford protein assay kit. The intracellular GPX activity was measured through a cellular glutathione peroxidase assay kit on the basis of the manufacturer’s instructions.

### Cell death and morphology determination

4T1 and B16 cells were preincubated in a 12-well plate at a density of 1.5 × 10^5^ cells and then co-incubated with different formulations (100 μM for 4T1 cells, 10 μM for B16 cells) at 37 °C. To monitor cell death and morphology, cells were visualized using the inverted microscope (Leica, DMi1, Germany). After incubation of 10 h, B16 cells were dyed with SYTOX green (10 nM) for 10 min at room temperature and imaged by fluorescence microscope (Leica, DMI6000B, Germany).

### LDH release assay

B16 cells were inoculated into 96-well plates at a density of 1.5 × 10^4^ cells per well overnight. Next, the culture medium was replaced by 150 μL of fresh culture medium containing different formulations (10 μM). After 9 h of incubation, the 96-well plates were centrifuged and the supernatants were collected. Then the amounts of LDH release were measured by an LDH cytotoxicity assay kit following the manufacturer’s instructions.

### Extracellular ATP detection

B16 cells were inoculated in the density of 1 × 10^5^ cells each well in 24-well plates overnight, and then treated with different formulations (10 μM). After 9 h incubation, the supernatants were collected by centrifugation and the release of ATP was detected by the luminescent ATP detection assay kit as described in the manufacture’s protocols.

### Immunofluorescence staining of ecto-CRT

The ecto-CRT exposure was detected by immunofluorescence staining. Briefly, confocal dishes-adhered B16 cells were treated with culture media containing different formulations of the same concentration (10 µM) at 37 °C for 10 h. Then cells were fixating by 4% paraformaldehyde, blocking by 1% bovine serum albumin (BSA), incubating with the primary antibody (Calreticulin Rabbit Monoclonal Antibody) at 4 °C overnight, staining with the secondary antibody (Goat Anti-Rabbit IgG (Alexa Fluor@488)) for 1 h and DAPI for 10 min at room temperature. The staining was imaged by CLSM.

### Statistical analysis

The experimental data were all statistically analyzed by GraphPad Prism (version 8.0.1). The data were presented in the form of mean ± SD. The comparison between groups was conducted by student’s two-tailed t-test. *P* < 0.05 was considered statistically significant.

## Supplementary Information

**Additional file 1.** Hypertoxic self-assembled peptide with dual functions of glutathione depletion and biosynthesis inhibition for selective tumor ferroptosis and pyroptosis.

## Data Availability

All data generated or analyzed during this study are included in this published article and its supplementary information file.

## Abbreviations

GSH: Glutathione
BSO: L-buthionine-sulfoximine
ROS: Reactive oxygen species
GST: Glutathione S-transferase
DEM: Diethyl maleate
ITCs: Isothiocyanates
GCL: Glutamate-cysteine ligase
GPX: Glutamate peroxidases
Fmoc: 9-Fluorenylmethyloxycarbonyl
TOF–MS: Time-of-flight mass spectrometry
TEM: Transmission electron microscopy
NPs: Nanoparticles
PBS: Phosphate buffered saline
HPLC: High-performance liquid chromatography
LC–MS: Liquid chromatography-mass spectrometry
IC_50_: Half-maximal inhibitory concentration
CLSM: Confocal laser scanning microscopy
RhoB: Rhodamine B
Fer-1: Ferrostatin-1
t-BuOOH: Tert-butylhydroperoxide
DCFH-DA: 2,7-Dichlorodihydrofluorescein diacetate
LDH: Lactate dehydrogenase
ATP: Adenosine triphosphate
GSDMD: Gasdermin D
GSDME: Gasdermin E
