# Supplementary material for: Hypertoxic self-assembled peptide with dual functions of glutathione depletion and biosynthesis inhibition for selective tumor ferroptosis and pyroptosis
Source: J Nanobiotechnology. 2022 Aug 31;20:390. doi: 10.1186/s12951-022-01604-5 (PMC9429723; doi:10.1186/s12951-022-01604-5)
Supplement: Supplementary file 1 — Additional file 1. Hypertoxic self-assembled peptide with dual functions of glutathione depletion and biosynthesis inhibition for selective tumor ferroptosis and pyroptosis. [file 12951_2022_1604_MOESM1_ESM.docx]

Additional file 1

Hypertoxic self-assembled peptide with dual functions of glutathione depletion and biosynthesis inhibition for selective tumor ferroptosis and pyroptosis

Yang Gao, Yun Li, Hongmei Cao, Haixue Jia, Dianyu Wang, Chunhua Ren, Zhongyan Wang, Cuihong Yang^*^ and Jianfeng Liu^*^

Key Laboratory of Radiopharmacokinetics for Innovative Drugs, Chinese Academy of Medical Sciences, and Institute of Radiation Medicine, Chinese Academy of Medical Sciences & Peking Union Medical College, Tianjin 300192, China

E-mail: liujianfeng@irm-cams.ac.cn (J. Liu), yangcuihong@irm-cams.ac.cn (C. Yang).

**1. Supplemental materials and methods**

**1.1. Materials**

Cystamine dihydrochloride, 1,6-hexanediamine and succinic anhydride were purchased from J&K Scientific Technology (Beijing, China). Fmoc-OSu, L-buthionine-sulfoximine (L-BSO) and 2-naphthaleneacetix acid were obtained from Shanghai Aladdin Biochemical Technology Co., Ltd. 2-chlorotrityl chloride resin, GSH (reduced) and Fmoc-amino acids were obtained from Nankai Resin Co. Ltd. (Tianjin, China), Energy Chemical Company (Shanghai, China) and GL Biochem (Shanghai, China), respectively. Rhodamine B, cell counting kit-8 (cck-8), Goat anti-rabbit IgG/HRP and Goat anti-mouse IgG/HRP were ordered from Solarbio Science & Technology Co., Ltd. (Beijing, China). 4T1, B16, NIH3T3, MCF-7, A549 and CT26 cell lines were all maintained in our lab. RPMI Medium 1640 basic, Dulbecco’s Modified Eagle’s Medium (DMEM), Fetal Bovine Serum (FBS) and 0.25% trypsin-EDTA were bought from Gibco (Beijing, China). GSH and GSSG assay kit, cellular glutathione peroxidase assay kit with NADPH, LDH cytotoxicity assay kit and detergent compatible bradford protein assay kit were purchased from Beyotime Biotechnology (Shanghai, China). Ferrostatin-1, Z-VAD-FMK, Ac-DEVD-CHO and Z-YVAD-FMK were acquired from MCE Co., Ltd. (Shanghai, China). BODIPY581/591 C11 and SYTOX green nucleic acid stain were bought from Thermo Fisher Scientific (Shanghai, China). Luminescent ATP detection assay kit, anti-GCLC antibody, anti-glutathione peroxidase 4 and anti-DFNA5/GSDME antibody were provided by Abcam (Shanghai, China). Cleaved caspase-3 (Asp175) antibody and beta-actin monoclonal antibody were obtained from Cell Signaling Technology (Shanghai, China) and Proteintech Group Inc. (Wuhan, China), respectively.

**1.2. Synthesis of Fmoc-CS**

The synthetic route of Fmoc-CS was as shown in Figure S1. Firstly, cystamine dihydrochloride (2.25 g, 10 mmol) and NaHCO_3_ (2.52 g, 30 mmol) were dissolved in 50 mL water. 50 mL of 1,4-dioxane was then added with stirring. Next, succinic anhydride (1.00 g, 10 mmol) was added to the above solution. And the mixture was stirred overnight. After the overnight reaction, NaHCO_3_ (0.84 g, 10 mmol) was added again. And 25 mL of 1,4-dioxane containing Fmoc-OSu (3.37 g, 10 mmol) was added to the above mixture dropwise. The reaction mixture continued to be stirred at room temperature overnight. After the reaction, the mixture was centrifuged to remove the insoluble substance and the supernatant was concentrated with the rotary evaporator. Then the concentrated solution was diluted with 300 mL of H_2_O and acidified to pH 2 with 1 M hydrochloric acid (HCl). Afterward, the white precipitate was collected by filtration and then dried in a vacuum. The obtained product was Fmoc-CS, which was directly used for peptide synthesis.

**1.3. Synthesis of Fmoc-BSO**

Fmoc-BSO was synthesized as Figure S2 displayed. BSO (1.11g, 5 mmol) and NaHCO_3_ (0.84 g, 10 mmol) were successively added to 50 mL water. After fully dissolved and being cooled to 0 °C in the ice bath, 20 mL of 1,4-dioxane containing Fmoc-OSu (1.685 g, 5 mmol) was slowly added to the reaction mixture. The reaction mixture was stirred for 2 hours at 0 °C and then stirred at room temperature overnight. After that, the reactants were also centrifuged, rotary steamed, acidified, filtered and vacuum dried. The resulting product was Fmoc-BSO, which was directly used without further purification.

**1.4. Synthesis of Fmoc-HDA**

Figure S3 showed the reaction process of Fmoc-HDA, which was similar to the reaction route of Fmoc-CS. 1,6-hexanediamine (1.3 mL, 10 mmol) and NaHCO_3_ (0.84 g, 10 mmol) were dissolved in 50 mL water. 50 mL of 1,4-dioxane was then added with stirring. Succinic anhydride (1.00 g, 10 mmol) was added to the above solution. And the mixture was stirred overnight. After the overnight reaction, N,N-Diisopropylethylamine (DIEA) was added to adjust the pH to 8-9. And Fmoc-OSu (3.37 g, 10 mmol) dissolving in 10 mL of DMF was added to the above solution dropwise. The reaction mixture continued to be stirred at room temperature overnight. After the reaction, the post-processing was the same as Fmoc-CS, including centrifugation, rotary evaporation, acidification, filtration and vacuum drying. The collected white powder was Fmoc-HDA without further purification.

**1.5. Synthesis of NSBSO, NCBSO and NS**

We synthesized the three peptide derivatives by standard solid phase peptide synthesis (SPPS) according to our previous literature [1]. 2-chlorotrityl chloride resin and corresponding N-Fmoc protected amino acids were used. It should be pointed out that in the synthesis of NSBSO and NCBSO, the first amino acid loaded on the C-terminus of the resin was Fmoc-BSO. Then disulfide bond or carbon-carbon bond were introduced by Fmoc-CS and Fmoc-HDA, respectively. And finally 2-naphthaleneacetix acid was coupled to the peptides. On the other hand, the synthesis of NS lacked the addition of Fmoc-BSO.

**1.6. Synthesis of RS and RC**

To synthesize the fluorescent analogs of NSBSO and NCBSO, we synthesized another two peptide derivatives: RS and RC through established SPPS protocol. At the last coupling step, Rhodamine B (RhoB) was utilized to replace 2-naphthaleneacetic acid attaching to the peptide. All the peptide derivatives were further purified by high-performance liquid chromatography (HPLC), dried by lyophilizer and confirmed by time-of-flight mass spectrometry (TOF-MS).

**1.7. Stability and compatibility detection of NSBSO**

The stability of NSBSO in 10% serum solution was analyzed by HPLC and the degradation rate was calculated. Specifically, NSBSO was dissolved in 10% serum solution at 400 μM and incubated at 37 °C. 100 μL samples were taken out at predetermined time points (1, 2, 4, 6, 8, 10, 12 and 24 h), and then 300 μL acetonitrile was added to precipitate the proteins in the sample at 4 °C for 15 min. Then, the sample was centrifuged at 10,000 rpm 10 min and the supernatant was collected for HPLC detection. The degradation rate of NSBSO was quantitatively analyzed by integrating the peak area of HPLC spectrum.

The blood cell compatibility of NSBSO was studied by hemolysis experiment. Specifically, samples were prepared according to the designed concentrations (62.5-4000 μM), and the PBS solution containing 0.1% Triton X-100 was used as the positive control. 400 μL samples with different NSBSO concentrations were gently mixed with 400 μL 2% red blood cell suspension in EP tubes, and all EP tubes were inverted and incubated at 37 °C for 3 h. The absorbance of 100 μL supernatant was measured by at 570 nm. The hemolysis rate was calculated by the following formula: Hemolysis rate (%) = (absorbance of sample-absorbance of negative control) / (absorbance of positive control-absorbance of negative control) × 100%.

**1.8. Cell culture**

The human breast adenocarcinoma cell line (MCF-7 cells), mouse melanoma cell line (B16 cells), human lung cancer line (A549 cells), mouse breast cancer cell line (4T1 cells), murine colon carcinoma cell line (CT26 cells) and mouse embryonic fibroblast cell line (3T3 cells) were used for research. MCF-7 and 3T3 cells were cultured in DMEM culture medium supplemented with 10% FBS and 1% penicillin/streptomycin (pen/strep) at 37 °C in 5% CO_2_. B16, A549, 4T1 and CT26 cells were cultured in RPMI Medium 1640 basic supplemented with 10% FBS and 1% penicillin/streptomycin (pen/strep) at 37 °C in 5% CO_2_.

**1.9. TEM and Bio-TEM sample preparation**

To prepare the TEM samples of NSBSO and NCBSO with or without GSH treatment *in vitro*, the solution of NSBSO and NCBSO (1 mM) was obtained as described in the main text, and then GSH (10 mM) was added to trigger the reaction. After that, the mixture (15 μL) was loaded on the carbon-coated copper grid. After being retained on the grid for 2 min, the excess sample was removed by filter paper carefully. Then 2% uranyl acetate solution (15 μL) was added to stain the grid, and removed by filter paper after 2 min. Afterward, the grid was placed in a desiccator to dry. To observe the distribution of self-assemblies and cell morphology changes in cells treated with NSBSO, Bio-TEM was used. At first, 10-cm dish-adhered 4T1 or B16 cells were incubated with NSBSO for different times (4 to 9 h) at a concentration of 100 μM or 10 μM, respectively. At the indicated time, cells were collected by trypsin digestion and washed using cold PBS and blood serum one after another. Then 2.5% glutaraldehyde was added slightly to cells for fixation overnight. Then cell samples were made into slices through the steps of staining, dehydration, embedding, sectioning and staining. And finally the sections were observed by 80 kV TEM (Hitachi, HT7800).

**1.10. Western blot analysis**

The protein expression of cells treated with NSBSO was assessed by western blot. Briefly, 4T1 or B16 cells were inoculated in 10-cm dishes for 24 h. Then the cells were incubated with different concentrations of NSBSO for different times at 37 °C. After that, the pretreated cells were gathered and the total protein was extracted from the cells by lysis buffer. The protein concentration was quantified by a detergent compatible bradford protein assay kit. Then lysates of each sample were run on 12% SDS-PAGE gels and transferred onto PVDF membranes, which were blocked with 5% non-fat milk in TBST for 1 h at room temperature, and then incubated with the corresponding primary antibody overnight at 4 °C. Immunoreacted bands were detected using secondary antibodies and developed using a Gel Imaging System (Bio-rad, Gel Doc XR).

**1.11. Annexin V-FITC/PI double staining analysis**

After incubation with 10 μM NSBSO for different times, the supernatant and cells were unified collected and centrifuged. After washed with PBS, the cell sedimentation was dispersed with 1 mL PBS with 2% FBS, followed by bathing in 37 °C water for 10 min. After washed with 100 μL 1× Binding Buffer once, the cells were counted and adjusted at a density of 1×10^6^/mL. Then 5 μL Annexin V-FITC was added to each sample for staining at room temperature for 20 min in the dark. And 5 μL PI was added and cells were stained for 10 min in the dark. Afterwards, 400 μL 1× Binding Buffer was added and mixed. The samples were examined with flow cytometry (Attune NxT).

**2. Supplemental figures**





**Figure S1.** The synthetic route of Fmoc-CS.





**Figure S2.** The synthetic route of Fmoc-BSO.





**Figure S3.** The synthetic route of Fmoc-HDA.


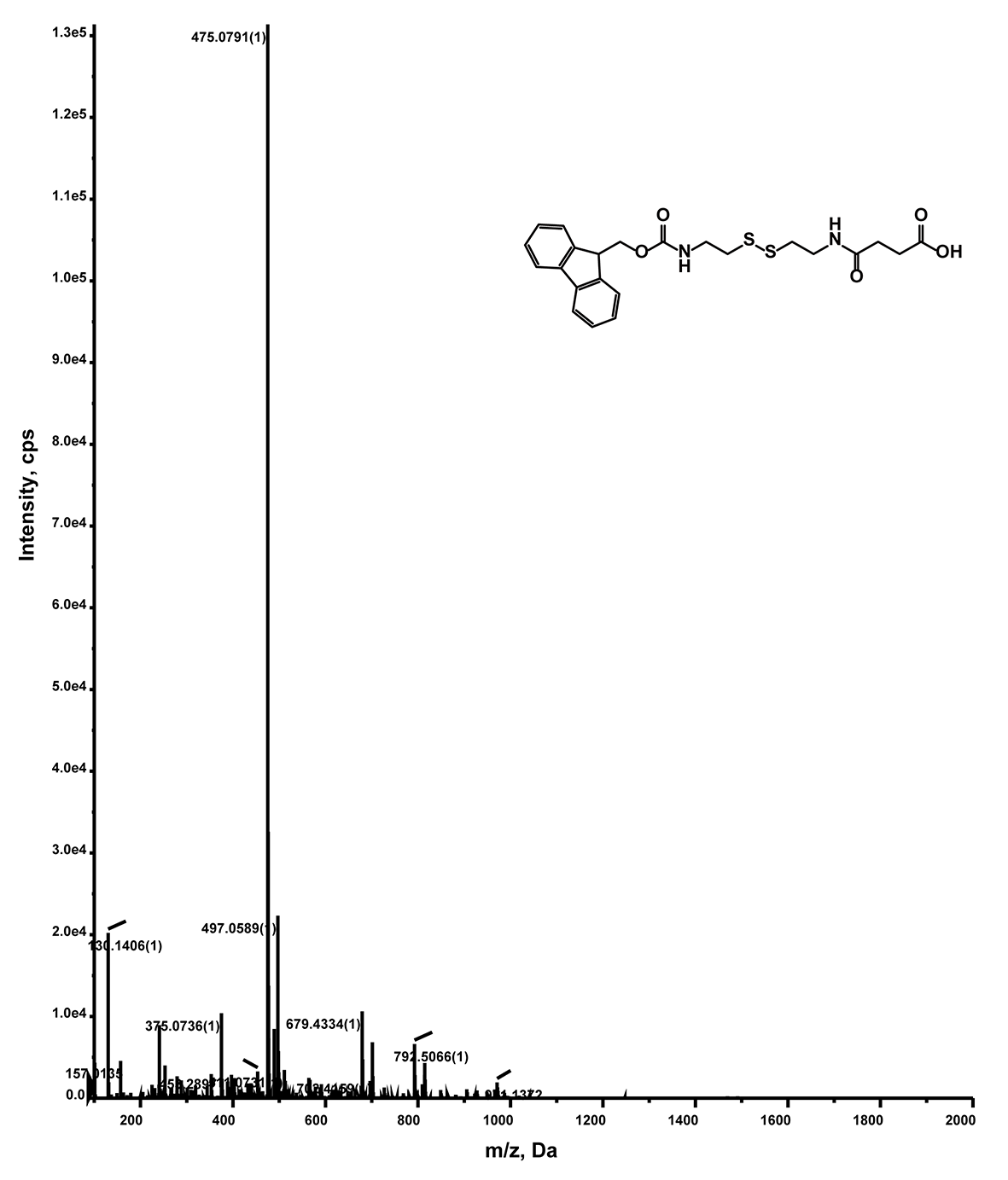


**Figure S4.** TOF-MS spectrum of Fmoc-CS.


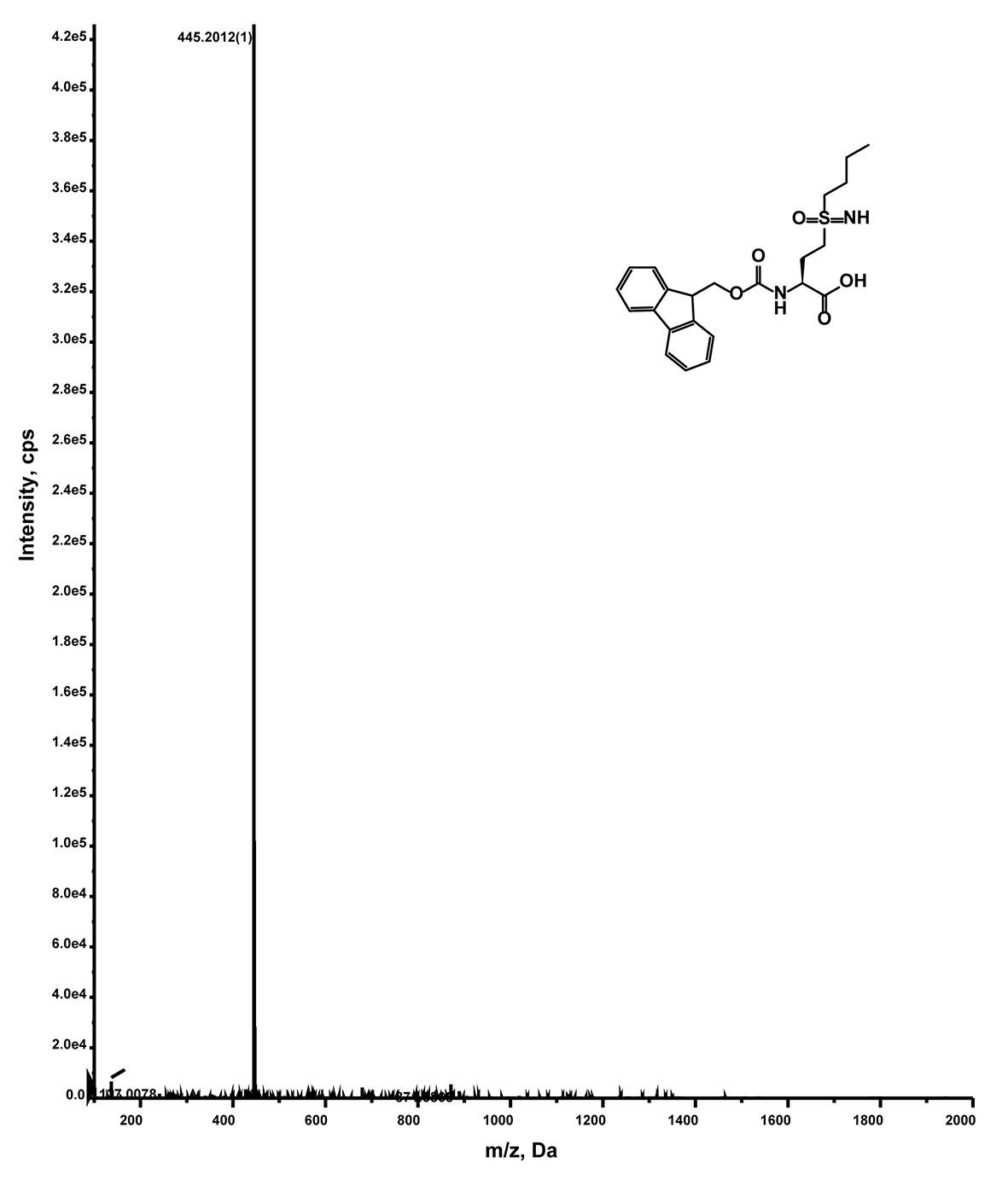


**Figure S5.** TOF-MS spectrum of Fmoc-BSO.


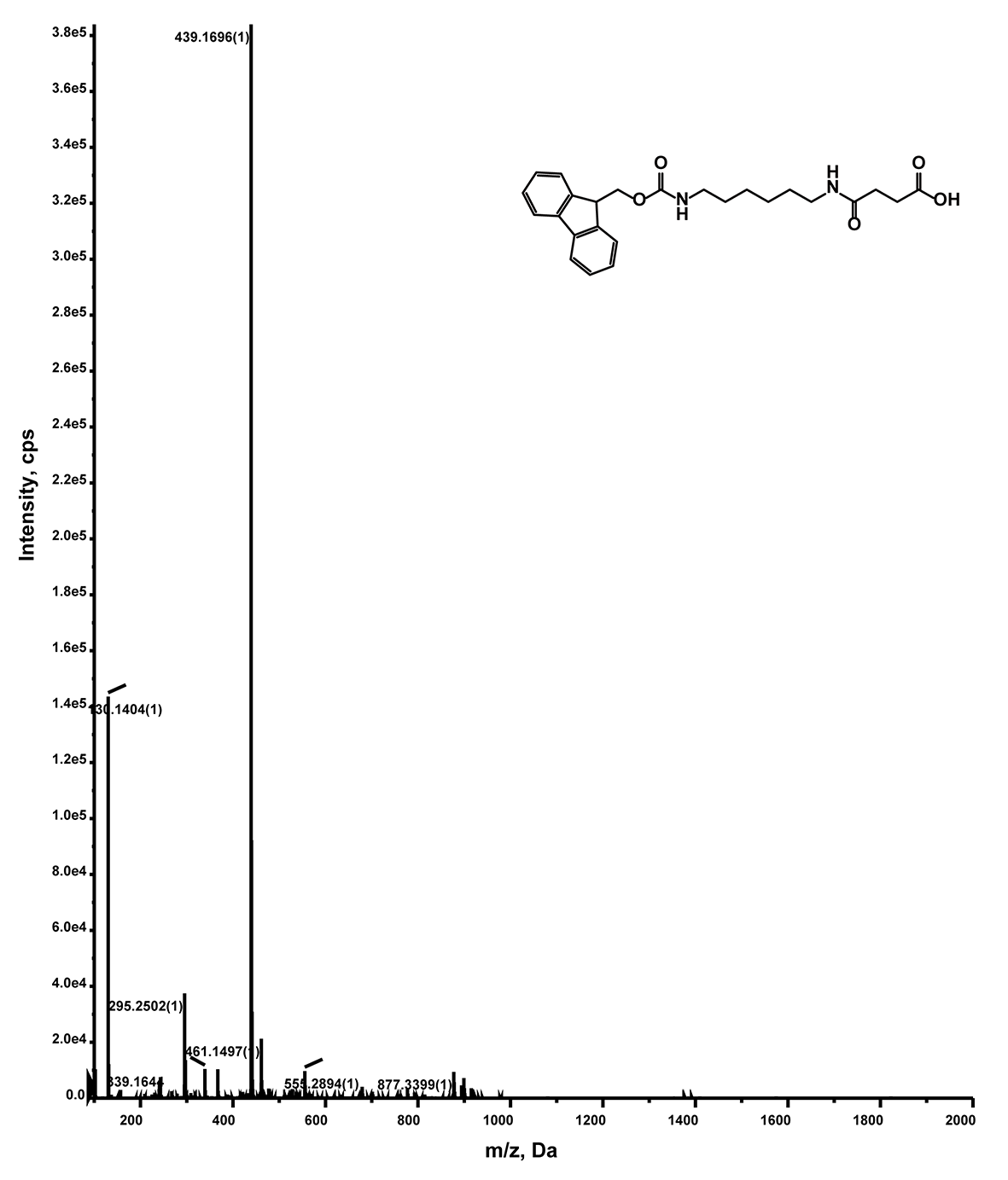


**Figure S6.** TOF-MS spectrum of Fmoc-HDA.


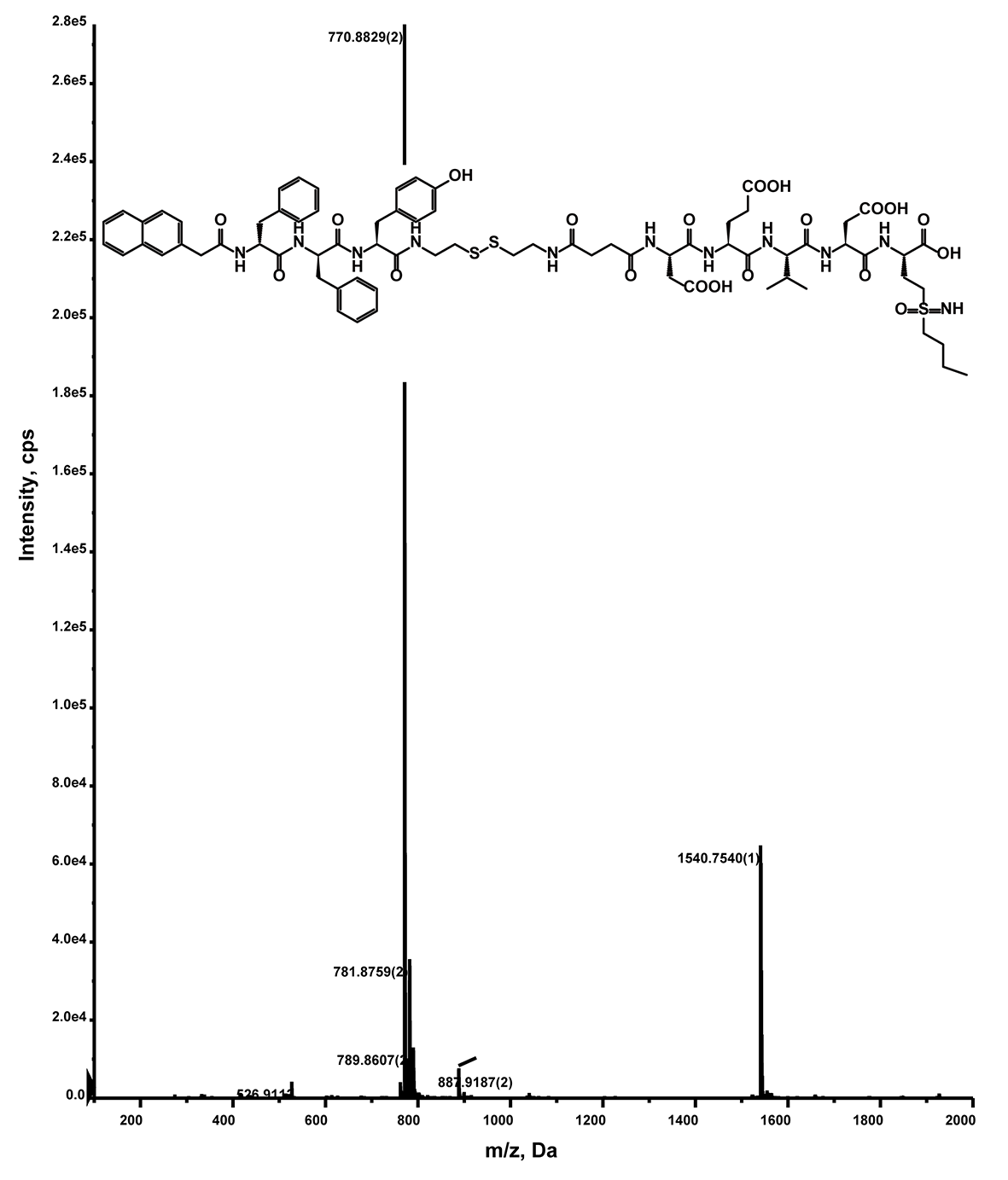


**Figure S7.** TOF-MS spectrum of Nap-^D^F^D^FY-CS-DEVD-BSO (NSBSO).


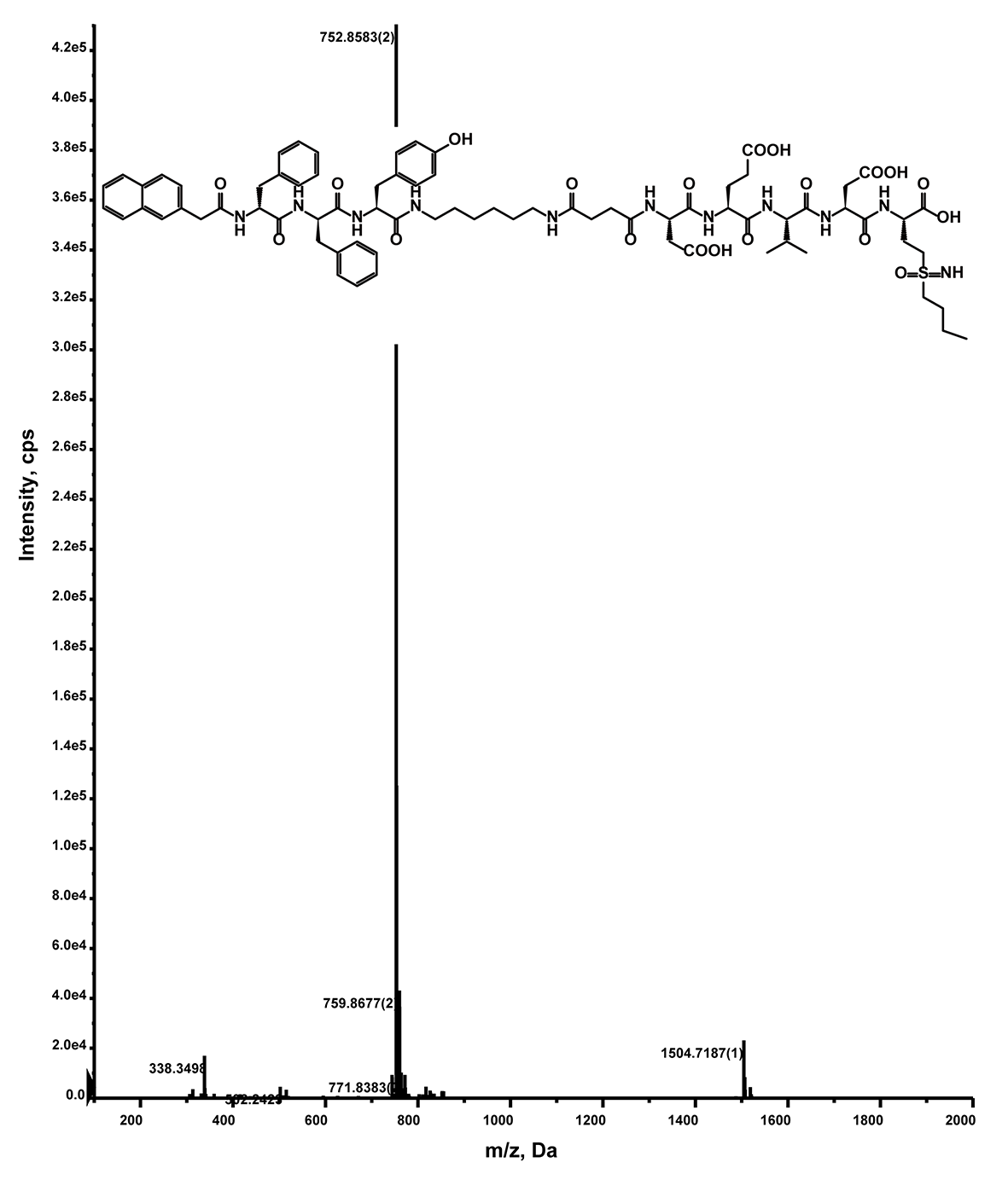


**Figure S8.** TOF-MS spectrum of Nap-^D^F^D^FY-HDA-DEVD-BSO (NCBSO).


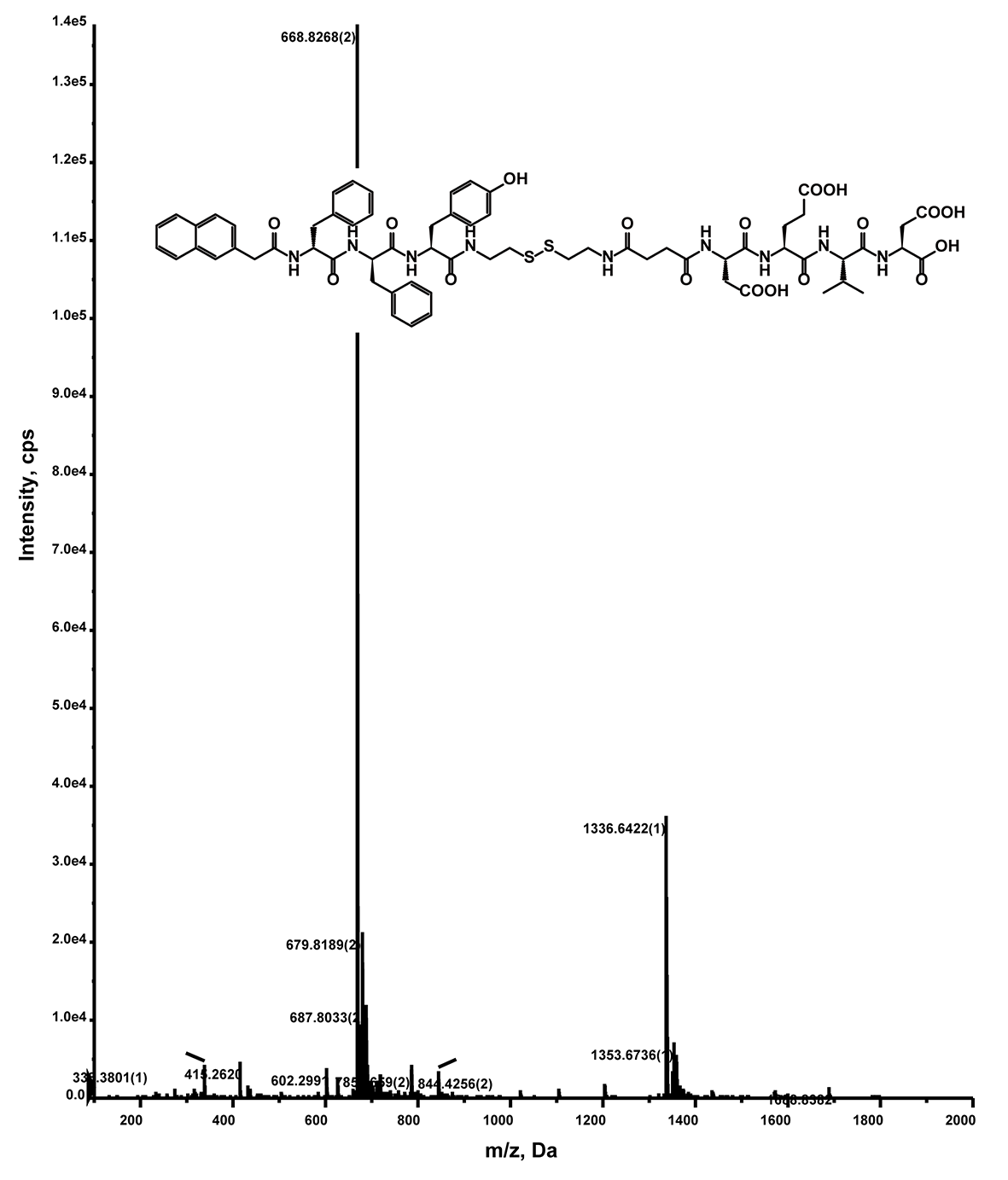


**Figure S9.** TOF-MS spectrum of Nap-^D^F^D^FY-CS-DEVD (NS).


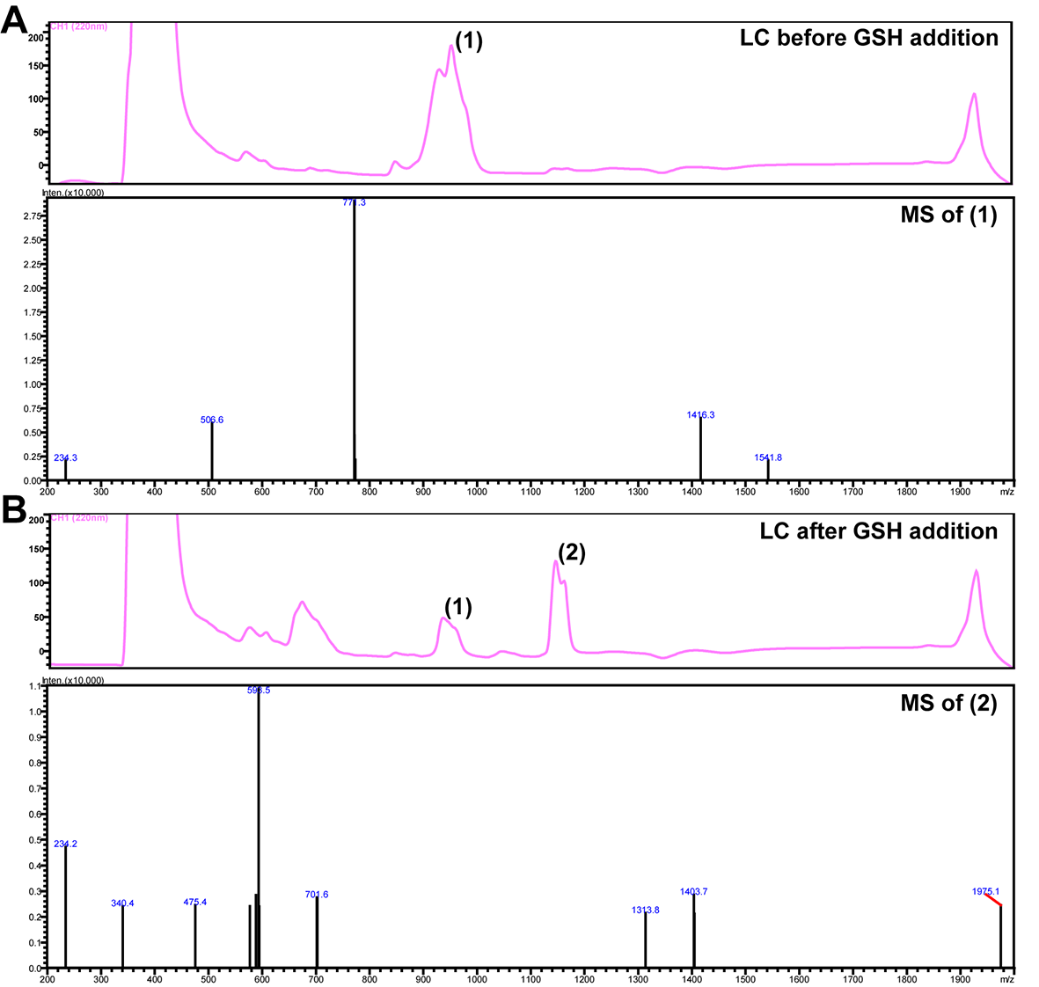


**Figure S10.** LC-MS spectra of NSBSO before (A) and after (B) the addition of GSH *in vitro*.





**Figure S11.** The conversion ratio of NSBSO by different concentrations of GSH at 37 °C within 6 hours.


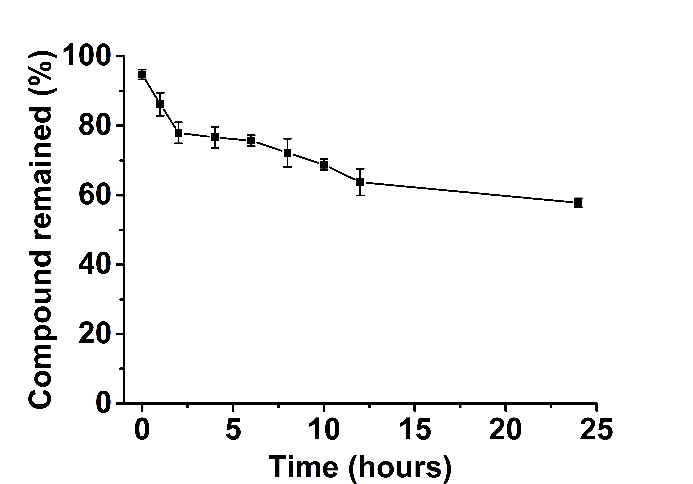


**Figure S12.** The stability of NSBSO in 10% serum at 400 μM within 24 h.


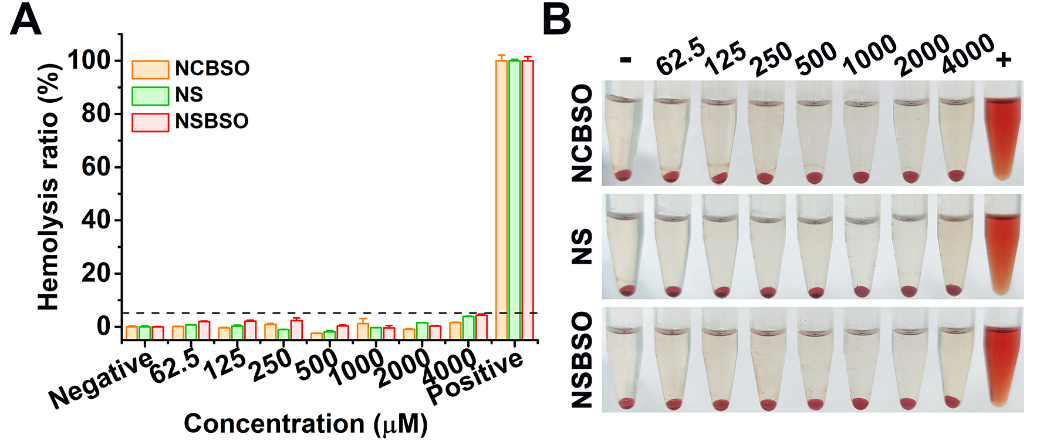


**Figure S13.** Hemolysis determination of NCBSO, NS and NSBSO at different concentrations at 37 °C for 3 h (A), and the photos of red blood cell mixtures after centrifugation (B).


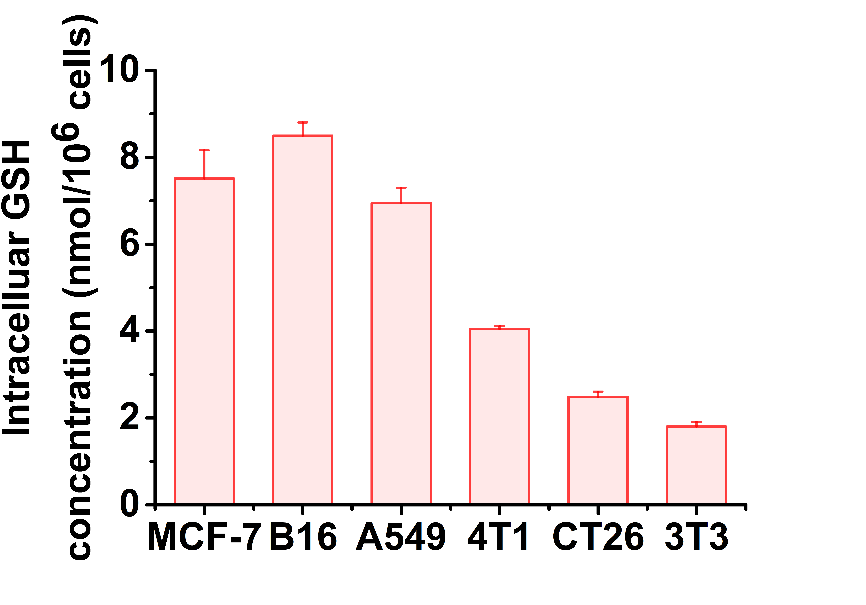


**Figure S14.** Intracellular GSH concentrations of different cells.





**Figure S15.** Cell viability of 3T3 cells after incubation with BSO, NCBSO, NS and NSBSO *in vitro* at different concentrations for 24 h.





**Figure S16.** Cytotoxicity of NSBSO and other formulations against 4T1 cells after incubation for 24 h.





**Figure S17.** Cytotoxicity of NSBSO and other formulations against B16 cells after incubation for 24 h.


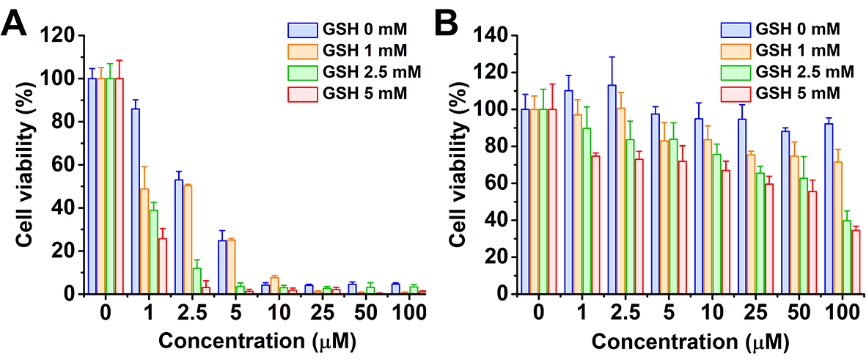


**Figure S18.** Cell viability of 4T1 cells (A) and CT26 cells (B) pretreated with different concentrations of GSH for 12 h and then treated with NSBSO for 24 h.


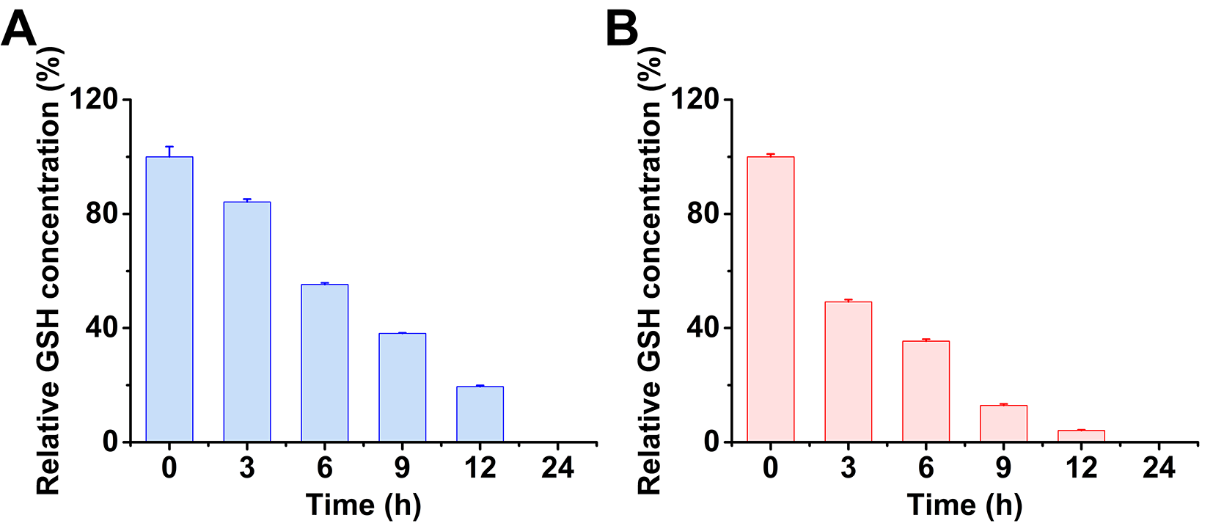


**Figure S19.** The relative GSH concentration in 4T1 cells (A) and B16 cells (B) upon incubation with GSH synthesis inhibitor (BSO, 1 mM) for different times.


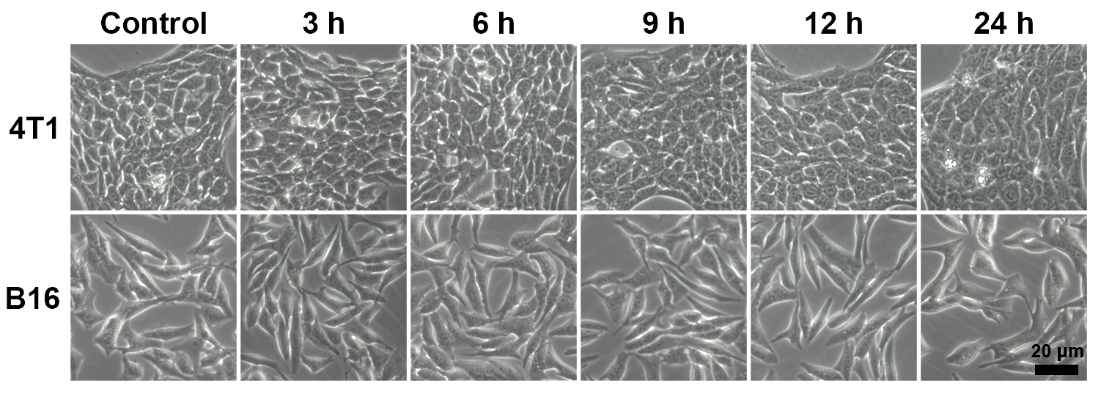


**Figure S20.** The cell morphology of 4T1 cells (A) and B16 cells (B) upon incubation with GSH synthesis inhibitor (BSO, 1 mM) for different times.


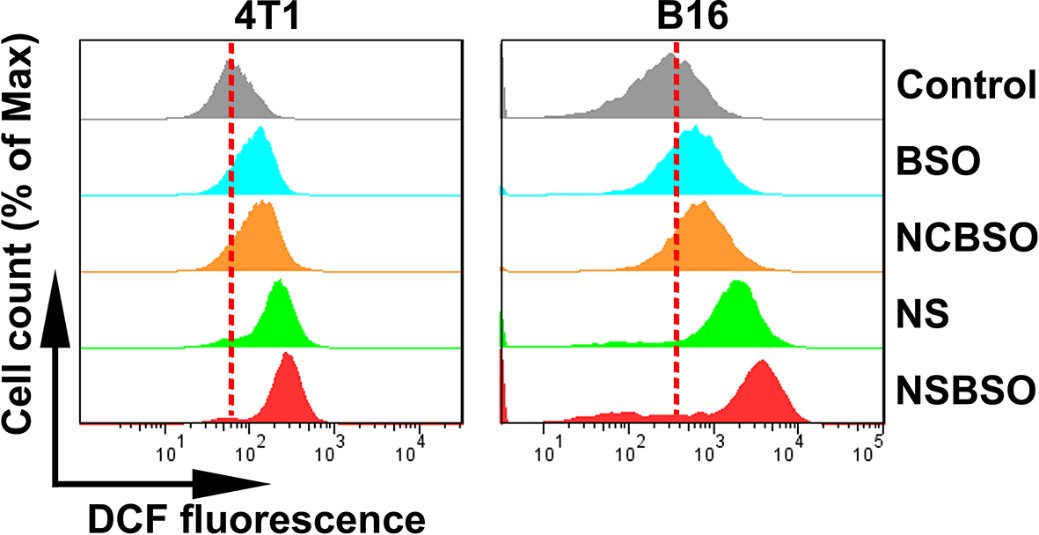


**Figure S21.** Flow cytometry analysis of DCFH-DA dye-stained 4T1 and B16 cells treated with different formulations for 12 h.


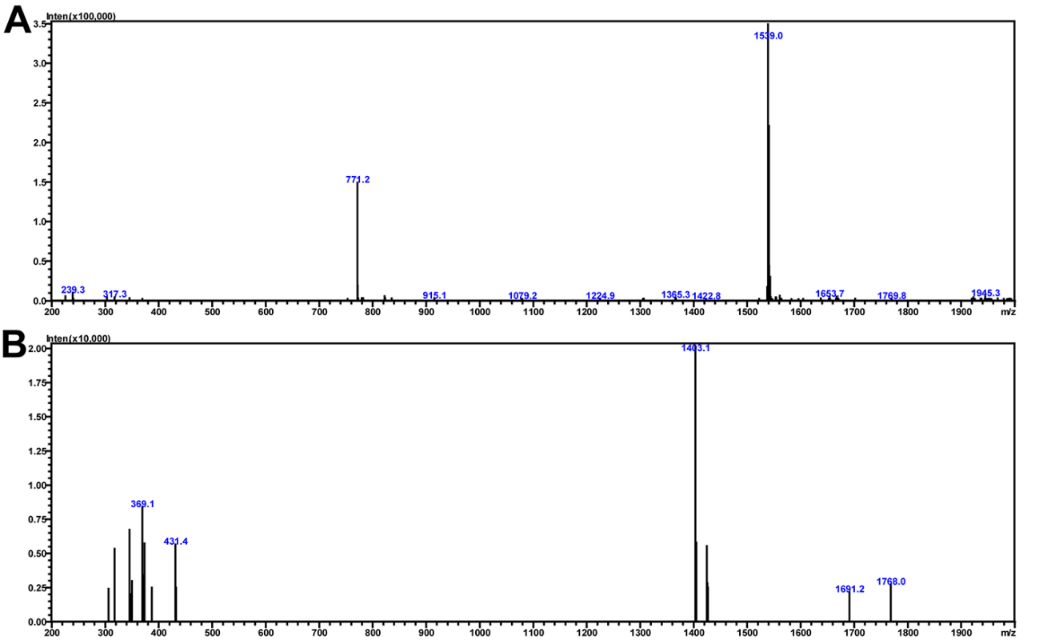


**Figure S22.** MS spectra of compound a (A) and compound b (B) in the culture medium and cell lysates after the co-incubation with NSBSO.


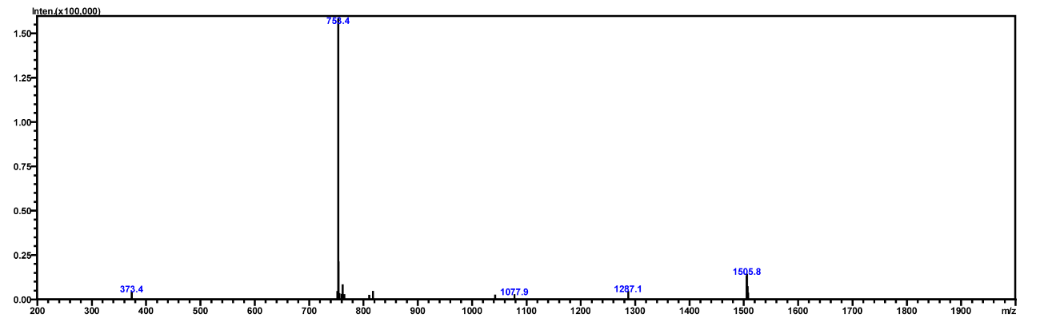


**Figure S23.** MS spectrum of compound c in the culture medium and cell lysates after the co-incubation with NSBSO or NCBSO.





**Figure S24.** Integral area of compound a, compound b and compound c in culture medium or cell lysates after incubating with 4T1 cells for 10 h.





**Figure S25.** Integral area of compound a, compound b and compound c in culture medium or cell lysates after incubating with B16 cells for 10 h.


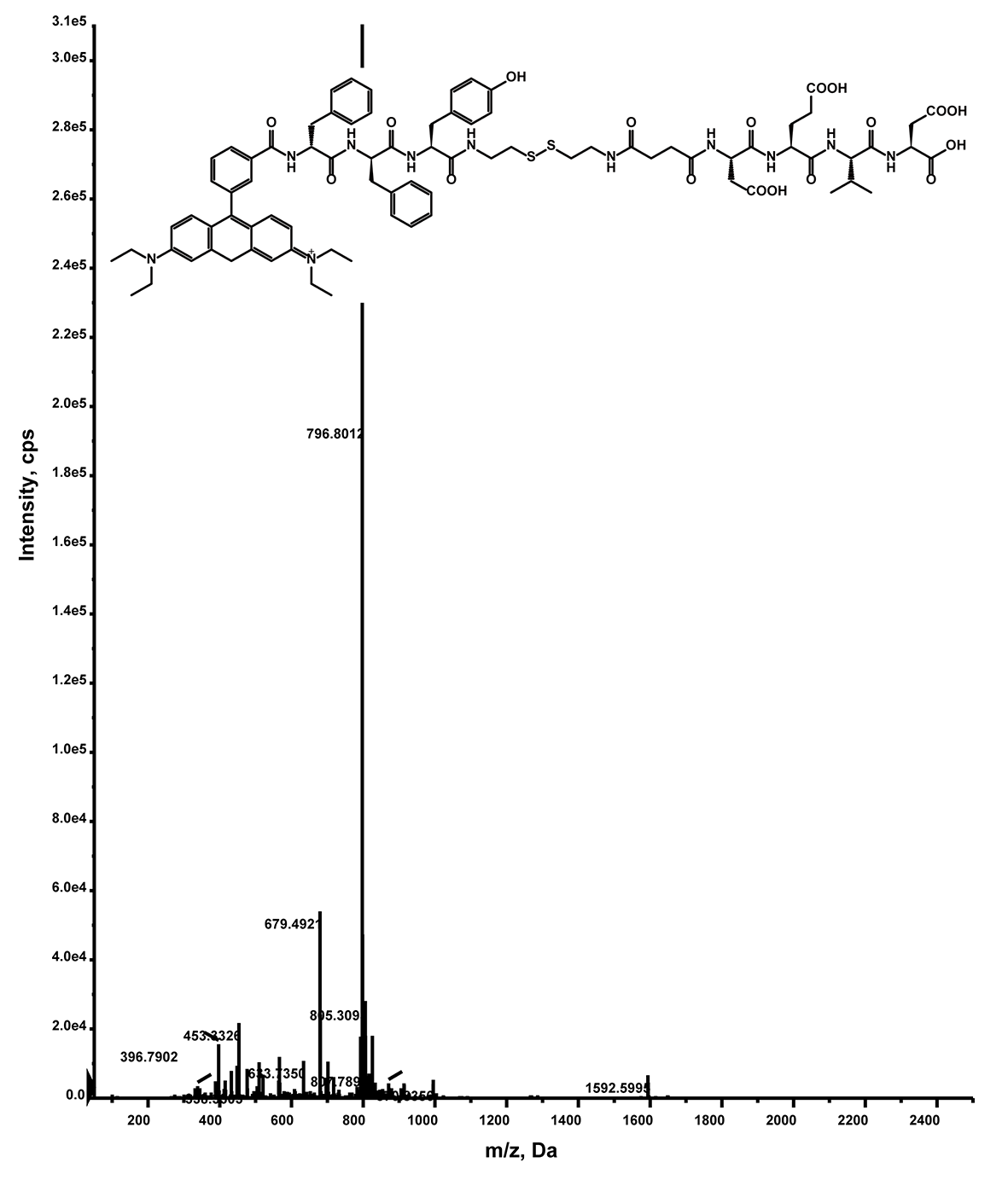


**Figure S26.** TOF-MS spectrum of RhoB-^D^F^D^FY-CS-DEVD (RS).


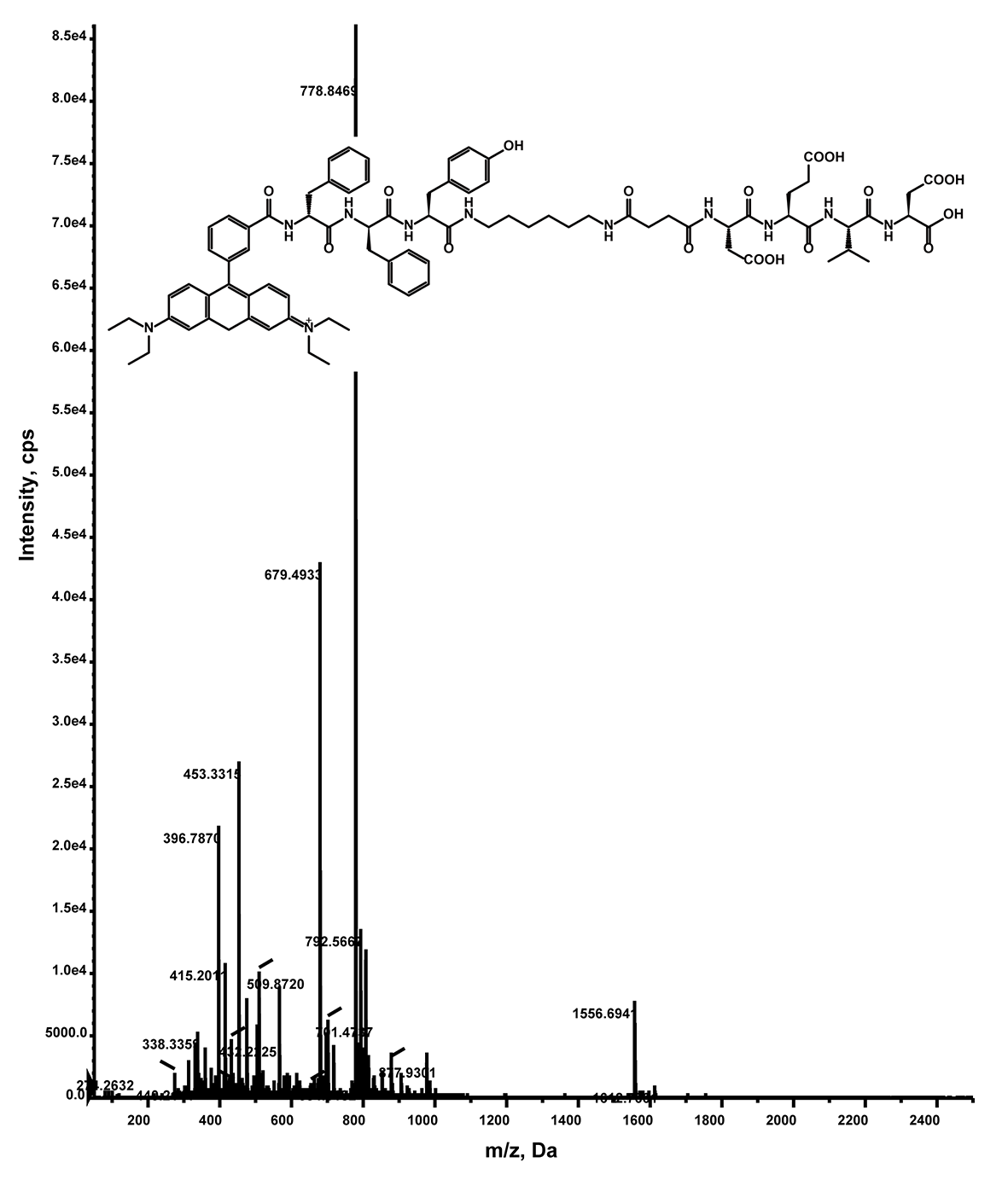


**Figure S27.** TOF-MS spectrum of RhoB-^D^F^D^FY-HDA-DEVD (RC).


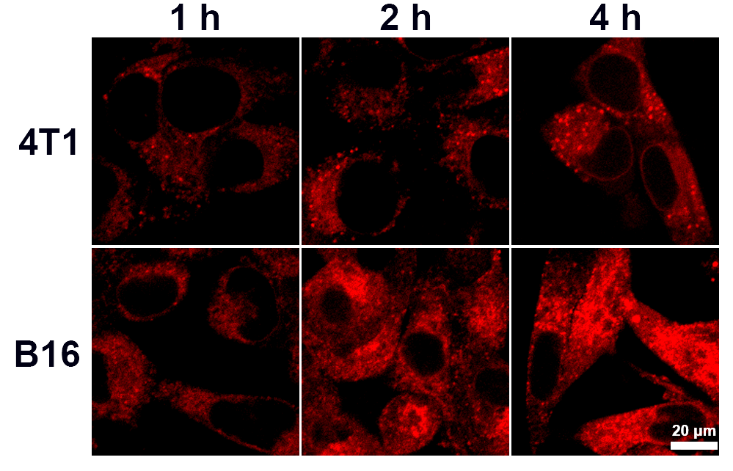


**Figure S28.** CLSM images of 4T1 and B16 cells treated with RC (100 μM) within 4 h.





**Figure S29.** Cell viability of 4T1 cells treated with NSBSO and ferroptosis inhibitor (Fer-1, 0.5 μM) for 24 h.





**Figure S30.** Cell viability of B16 cells treated with NSBSO and ferroptosis inhibitor (Fer-1, 0.5 μM) for 12 h.





**Figure S31.** Cell viability of 4T1 cells treated with NSBSO (5 μM) and apoptosis inhibitor (Z-VAD-FMK) for 24 h.


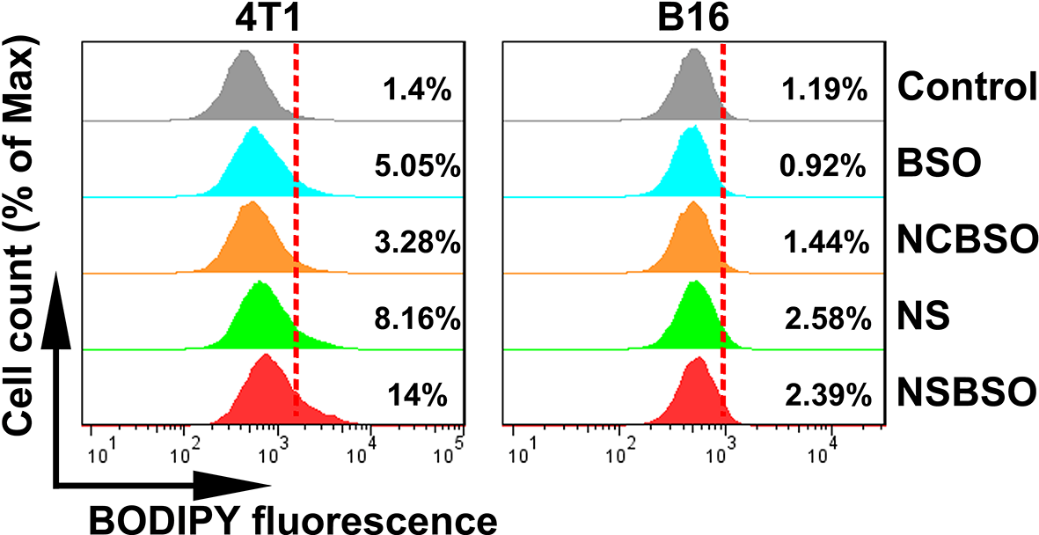


**Figure S32.** Flow cytometry analysis of BODIPY dye-stained 4T1 and B16 cells treated with different formulations (10 μM, 10 h)


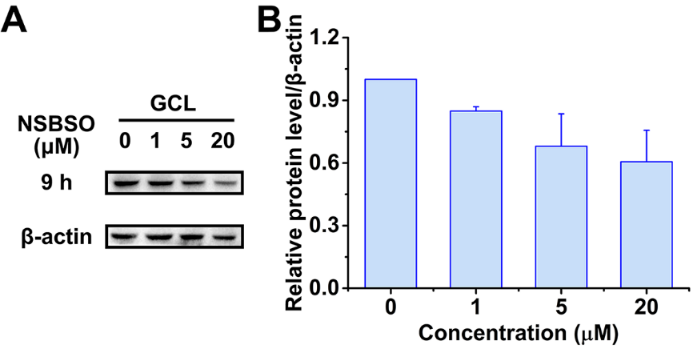


**Figure S33.** The expression level of GCL in 4T1 cells treated with different concentrations of NSBSO for 9 h (A) and the semi-quantification of the western blot results (B).


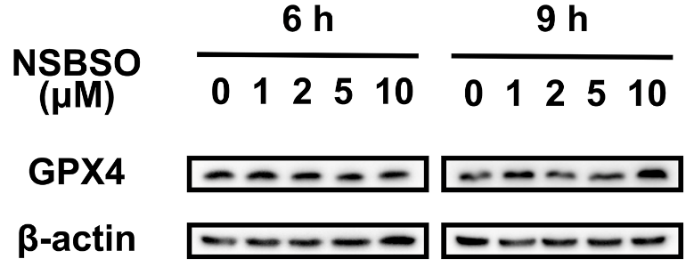


**Figure S34.** The expression level of GPX4 in B16 cells treated with different times and different concentrations of NSBSO.


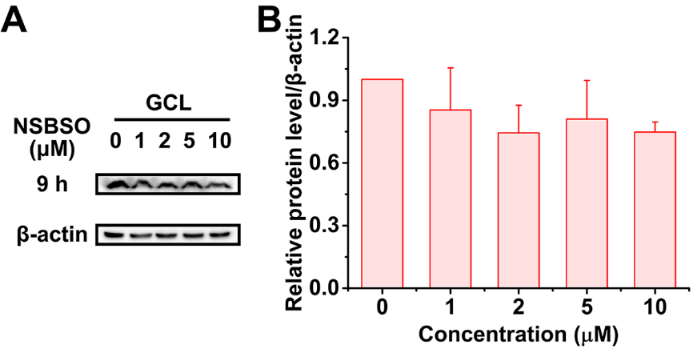


**Figure S35.** The expression level of GCL in B16 cells treated with different concentrations of NSBSO for 9 h (A) and the semi-quantification of the western blot results (B).


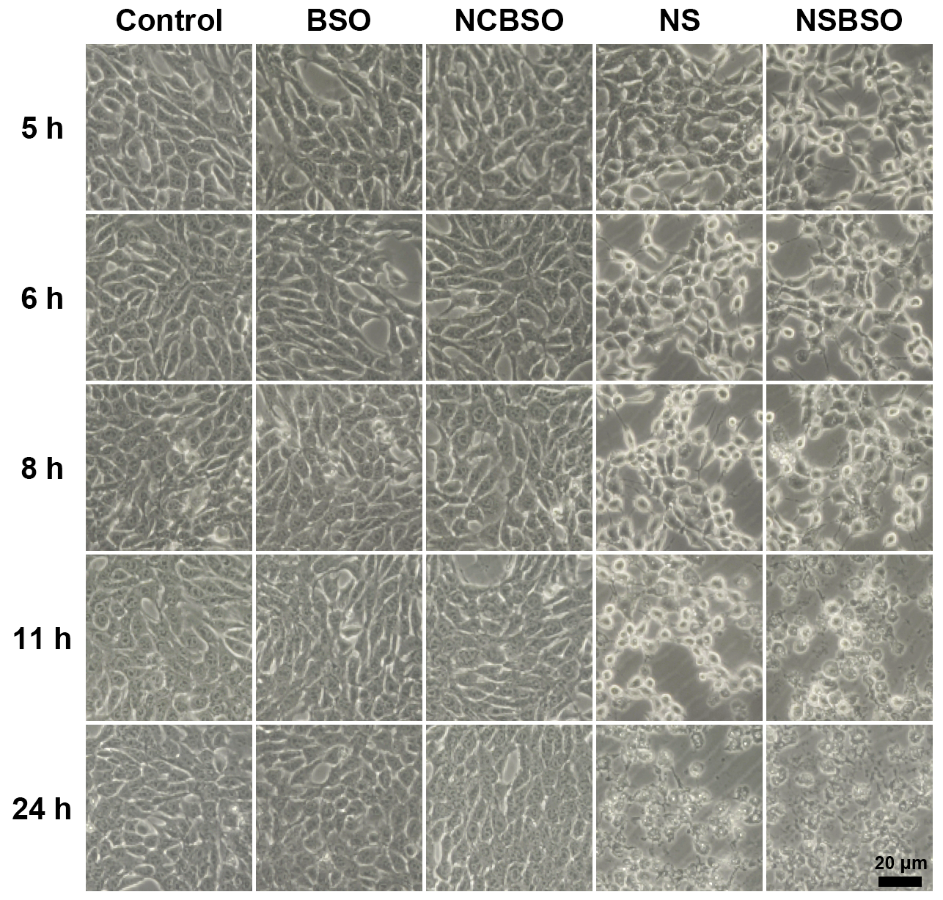


**Figure S36.** Representative images of the morphology of 4T1 cells treated with different formulations (100 μM).





**Figure S37.** Cell viability of B16 cells treated with NSBSO, caspase 1 inhibitor (Z-YVAD-FMK) and caspase 3 inhibitor (Ac-DEVD-CHO) for 12 h.


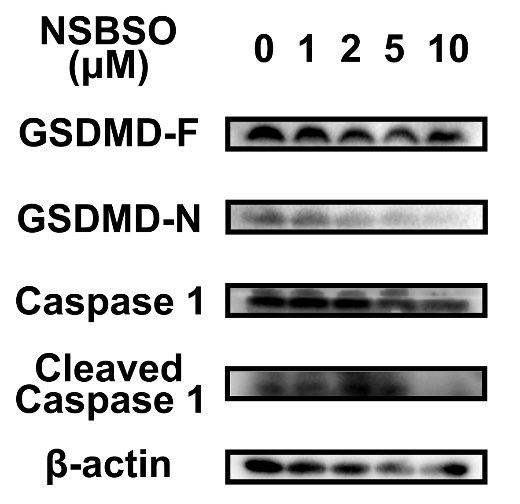


**Figure S38.** The expression level of GSDMD-F, GSDMD-N, Caspase 1 and Cleaved Caspase 1 in B16 cells treated with different concentrations of NSBSO for 6 h.


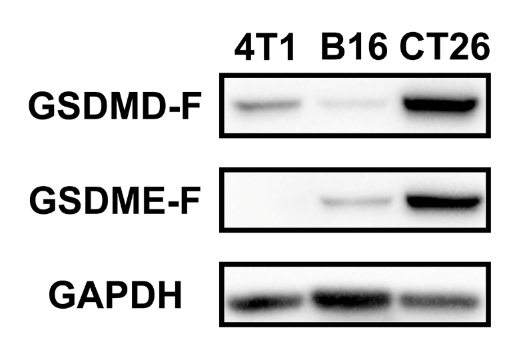


**Figure S39.** The expression level of GSDMD-F and GSDME-F in untreated 4T1, B16 and CT26 cells.


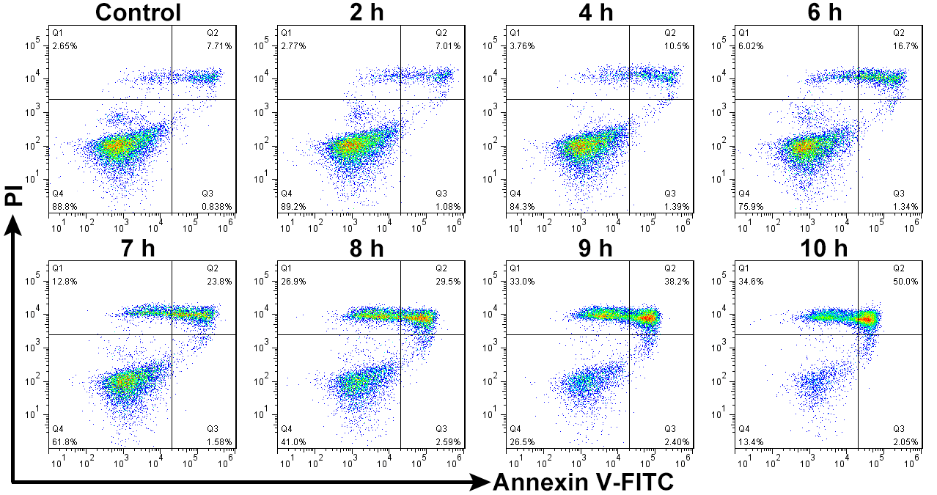


**Figure S40.** Annexin V/PI double staining analysis of B16 cells after 10 μM NSBSO treatment within 10 h.


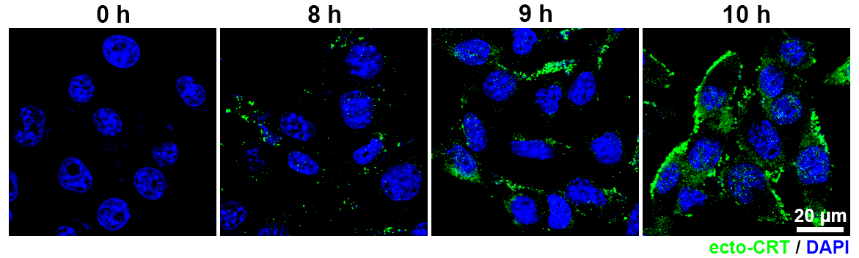


**Figure S41.** Immunofluorescence images of surface-exposed calreticulin (ecto-CRT) in B16 cells treated with NSBSO (10 μM) for different times.

**References**

1. Gao Y, Zhang C, Chang J, Yang C, Liu J, Fan S, Ren C. Enzyme-instructed self-assembly of a novel histone deacetylase inhibitor with enhanced selectivity and anticancer efficiency. Biomater Sci. 2019;7:1477-85.
